# Advances in Antimicrobial Applications of Ag, Cu, and AgCu Nanoparticle-Doped Polymeric Composite Materials: A Comprehensive Review

**DOI:** 10.1021/acsnano.5c08822

**Published:** 2025-08-25

**Authors:** Joana Galhano, José Luis Capelo-Martinez, Julia Lorenzo, Carlos Lodeiro, Elisabete Oliveira

**Affiliations:** † BIOSCOPE Research Group, LAQV-REQUIMTE, Chemistry Department, NOVA School of Science and Technology, FCT NOVA, 119482 Universidade NOVA de Lisboa, Caparica 2829-516, Portugal; ‡ PROTEOMASS Scientific Society, Caparica 2825-466, Portugal; § Institut de Biotecnologia i Biomedicina, Departament de Bioquímica i de Biologia Molecular, 16719 Universitat Autonoma de Barcelona, Bellaterra, Barcelona 08193, Spain; ∥ School of Science, Psychology, Arts & Humanities, Computing, Engineering and Sport, Canterbury CT1 1QU, United Kingdom

**Keywords:** silver−copper nanoparticles, antimicrobial activity, bimetallic nanoparticles, silver nanoparticles, copper nanoparticles, healthcare applications, food packaging, polymeric matrixes, nanoparticle-doped polymers

## Abstract

The growing challenge of antimicrobial resistance (AMR) has driven the search for alternative strategies to conventional antibiotics, with metallic nanoparticles (NPs), particularly silver (Ag), copper (Cu), and their bimetallic hybrids (AgCu), emerging as promising candidates. These nanoparticles exhibit strong, multifaceted antimicrobial activity and, when integrated into polymeric matrices, form composite materials that offer enhanced stability, controlled release, and broad applicability across healthcare, food safety, and industrial sectors. This review highlights the major synthetic routes for the production of Ag, Cu, and AgCu nanoparticles and discusses their integration into polymeric systems. Furthermore, the antimicrobial mechanisms of these nanomaterials are explored. Finally, the review emphasizes the potential of these nanocomposites to serve as next-generation antimicrobial surfaces, wound dressings, and functional materials, while addressing key synthesis challenges and proposing future research directions to optimize their performance and expand their applications.

## Graphical abstract

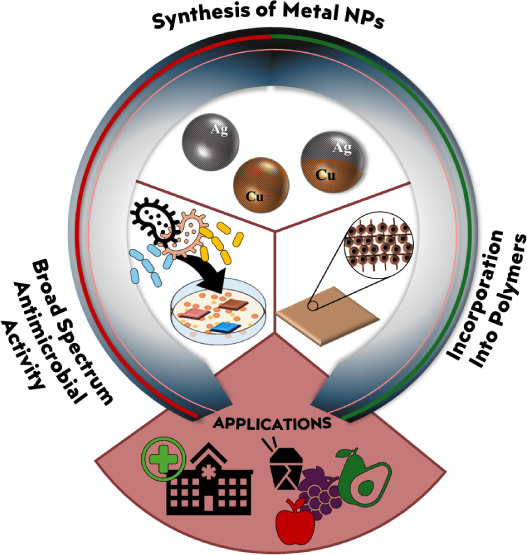

## 1 Introduction

With increasing rates of antimicrobial resistance (AMR) in both nosocomial and domestic settings, developing materials that could hinder this progress is of utmost importance. AMR is widely recognized as a leading global public health and development issue. Bacterial-related AMR has resulted in an estimated 1.27 million deaths in 2019 alone.^1^ The production and development of new antimicrobial drugs, such as new antibiotics, is not a workable alternative nowadays due to the lengthy research and trial processes, and the excessive costs associated with these processes. On average, a new antimicrobial drug takes roughly 10 years from the research stage to being marketed, with only 12.5% of the submitted drugs moving successfully from the registration phase.^2^ This period is not workable to successfully hinder AMR progression, as resistance mechanisms spread at a much faster rate. Instead, more practical approaches, based on prevention instead of treatment, have been developed in the last few years, aiming to develop products that can be integrated into a simpler way in society, with the goal of preventing the occurrence of infections in the first place.^3,4^

In this context, nanomaterials have emerged as a strong alternative material.^5^ Metallic nanoparticles, particularly silver nanoparticles (AgNPs), have gained significant attention due to their bioactive characteristics. AgNPs have already found applications in direct consumer products such as cosmetics, deodorants, and antiaging creams,^6^ due to their broad-spectrum antimicrobial activity.^7^ Copper nanoparticles (CuNPs) have also been extensively studied, with their bioactive properties receiving significant attention, although with less representativity in a commercial setting.^8^ Additionally, the combination of these two types of metals, yielding bimetallic nanoparticles (AgCu NPs), is also an explored strategy, although not as much as their monometallic counterparts. Nevertheless, bimetallic silver–copper nanoparticles (AgCu NPs) have already demonstrated significant promise due to the synergistic interactions between the two metals, enhancing the effects of their monometallic components.^9^

Nanomaterials benefit further from their incorporation onto solid-supported matrices, such as polymeric ones, allowing for their usage as high-risk surfaces, coatings, or membranes. Through the production of nanoparticle-doped polymers, applications as diverse as clinical or food-related become available at a much faster time rate than if applied directly as medications or for human consumption. This is yet another strategy to prevent the spread of resistance mechanisms within microorganism communities through a preventative mechanism, instead of the typically employed theragnostic perspective.

Considering these points, this review aims to provide a comprehensive analysis of the various research stages from nanoparticle synthesis and introduction into polymeric matrixes to their specific applications and bioactive mechanisms. Figure 1 summarizes the major points discussed in this review.

**Figure 1:**
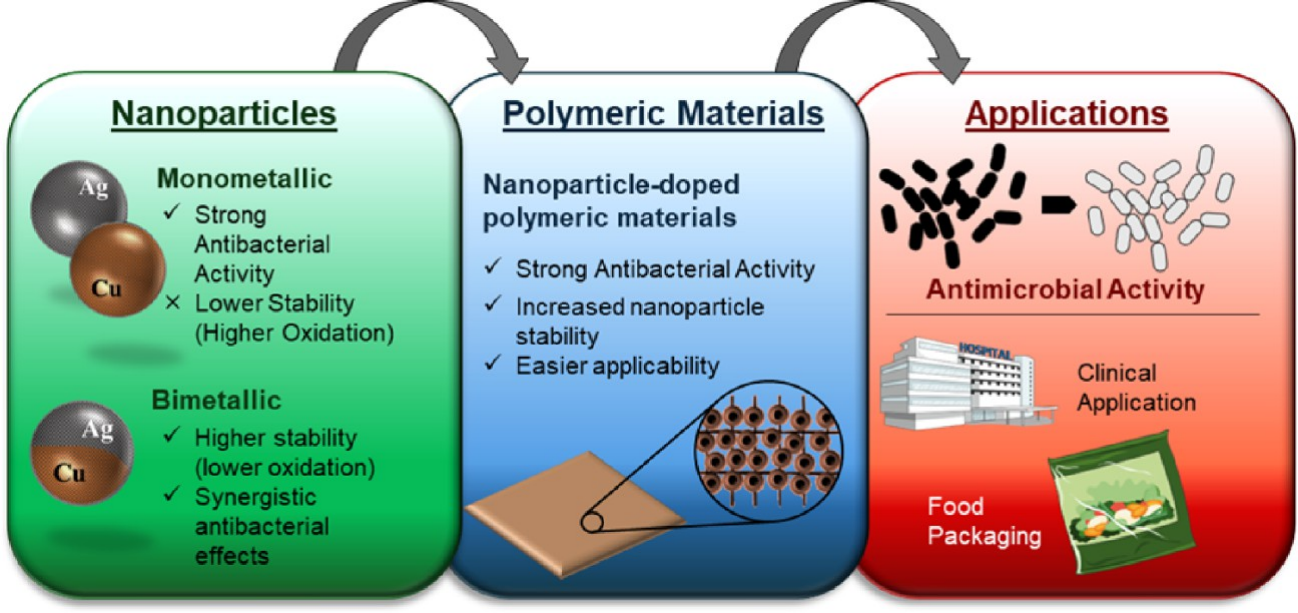
General description of the topics detailed in this review.

## 2 Selection Criteria

A systematic literature review was conducted in PubMed, Web of Science, and Scopus databases, with a time frame displaying relevant articles from January 1, 2015 onward. The major keywords used for the advanced search options to compile relevant articles to present in Tables 1–3 of this review were “nanoparticles”, “silver nanoparticles”, “silver nanoparticles AND polymers”, “copper nanoparticles”, “copper nanoparticles AND polymers”, “antimicrobial AND silver nanoparticles”, “antimicrobial AND copper nanoparticles”, and “antimicrobial AND silver–copper nanoparticles”. Additional keywords were applied when necessary for a more detailed subject. The obtained articles were sorted by relevance in the respective databases, and an initial analysis was conducted through the assessment of titles and abstracts, followed by an in-depth complete analysis of the full manuscripts presented.

**Table 1:** Common Methods for the Synthesis of AgNPs

Synthetic Route: Chemical					
Method	Ag Precursor	Reducing Agent	Capping Agent	Size (nm)	ref
Chemical Reduction	AgNO_3_	Sodium citrate and tannic acid	-	≃30	^35^
	AgNO_3_	Sodium borohydrate	-	≃20	^35^
	AgNO_3_	*N,N*-dimethylformamide	3-aminopropyltrimethoxysilane	≃7 to 20	^36^
	AgNO_3_	Tannic acid and sodium citrate	-	≃4 to 40	^30^
Photochemical Reduction	AgNO_3_	Sodium citrate	Sodium citrate	≃44	^37^
Microwave-assisted	AgNO_3_	Apple extract	-	≃22	^38^
Sonochemical method	AgNO_3_	Glucose	Gelatin	≃5.3	^39^

**Synthetic Route:** Electrochemical				
Ag Precursor	Stabilizer	Electrodes	Parameters	ref
AgNO_3_	PEG	Platinum rod	2.0, 2.9, 3.7 mA·cm^–2^	^40^
AgNO_3_	Citrate	Silver electrode	*U* = 12 V	^41^
Sacrificial anode	PVP	Silver rod	*U* = 10 V; 60 °C	^42^
AgNO_3_	PVP	Platinum sheets	100 mA·cm^–2^	^43^
Sacrificial anode	-	Silver rods	10 mA·cm^–2^	^44^

**Synthetic Route:** Physical				
Method	Ag precursor	Size (nm)	Conditions	ref
Ball/bead milling	Silver powder	4–8	Ar atmosphere, below –160 °C	^45^
Electrical arc-discharge	Silver wire	-	Deionized water, 25 °C	^46^
Laser ablation	Silver plate	20–50	Laser pulse, PVP as a stabilizing agent	^47^
Physical vapor condensation	Silver wire	≃20	Fructose as a stabilizing agent, rapid cooling	^48^

**Synthetic Route:** Biological					
Microorganism		Ag precursor	Size (nm)	Bioactive compound	ref
Bacteria	*Streptomyces violaceus*	AgNO_3_	10–60	Exopolysaccharide	^49^
	*Pseudomonas*	AgNO_3_	10–40	Aliphatic and aromatic amines	^50^
Algae	*Padina*	AgNO_3_	≃40	Marine extract	^51^
Fungi	*Fusarium oxysporum*	AgNO_3_	20–40	Proteins	^52^
	*Aspergillus tamarii*	AgNO_3_	≃4	NADH-dependent nitrate reductase	^53^
Plant	*Abelmoschus esculentus*	AgNO_3_	5–25	Flower extract	^54^

## 3 Design and Application of Antimicrobial Nanomaterials

Silver- and copper-containing nanomaterials (NMs) have advantages over the more commonly used gold- and bismuth-based materials. Bismuth-based nanomaterials (Bi-NMs) have recently emerged as effective alternatives for combating bacterial infections, owing to their abundant reserves, low cost, unique semimetallic properties, and high stability. Unlike noble metal nanomaterials such as gold (Au), Bi-NMs exhibit considerable antibacterial activity through mechanisms such as photothermal conversion and photocatalysis, particularly when stimulated by light irradiation.^10^ These processes generate reactive oxygen species (ROS) that damage bacterial cell membranes and biofilms and disrupt DNA synthesis. However, despite these promising features, Bi-NMs face limitations in their antibacterial efficiency due to low photothermal conversion and photocatalytic abilities when used as single-material nanostructures.

In contrast, silver (Ag) and copper (Cu) nanomaterials present distinct advantages in antimicrobial applications. Silver ions are highly effective against a broad range of bacteria and fungi, working through membrane disruption and DNA interference. Copper, known for its redox activity, contributes to the generation of ROS, significantly enhancing the antibacterial efficiency. Unlike Bi-NMs, which rely heavily on light activation for full antibacterial activity, Ag and Cu nanomaterials offer intrinsic antibacterial properties that are effective even without external stimuli. This makes them more versatile and easier to apply across various settings including biomedical, environmental, and agricultural applications. While Bi-NMs continue to show promise, the development of silver- and copper-based nanomaterials advances further, offering more reliable and cost-effective solutions to combat bacterial resistance and infection. The combination of their cost-effectiveness, broad-spectrum activity, and ease of functionalization makes Ag and Cu nanomaterials the preferred choice over Bi-NMs in many antibacterial strategies.

The promising role of nanoparticles as antimicrobial agents is clear; however, it does not come without associated hindrances. One of the most crucial points to consider in the application of nanomaterials as antimicrobial agents is the development of a well-defined synthetic protocol that yields uniform, monodisperse nanoparticles consistently. Particularly, if these are to be administered in a clinical setting, with potential for human application, it is of utmost importance that the nanoparticles meet a series of prerequisites to ensure their safety.^11^ As these particles and their effects are tightly correlated to the shape, size, and possible functionalization systems, a synthetic route that yields nonhomogeneous particles might cause unknown negative responses that decrease the efficacy of the material itself and introduce additional risks to its use. To reduce such risks, production protocols must be evaluated through the Good Manufacturing Practice (GMP) guidelines^12^ and a detailed workflow that focuses not only on the conscientious design of the nanoparticles but also on thorough characterization and prediction of bioactive profiles, whether through *in silico* or *in vivo* models.^13^ These guidelines should be implemented from the early stages of research and development to ensure that all results obtained from experimental settings are translatable into potential commercialized materials, with a possible direct application in society.

A specific requirement for this passage is the scalability of the production processes, which should be considered through a multidisciplinary viewpoint to ensure that the properties and overall quality of the materials produced at a bench level are the same as those produced by large-scale industrial processes, ensuring similar bioactive responses and mechanisms. Additionally, sustainability and practicability for both scale-up and bench-level research and development of the synthetic processes should also be considered when developing or applying already existent protocols. However, a significant gap still exists between bench-level studies and their scaled-up industrial counterparts, despite the growing demand for such advancements due to the enhanced bioactive properties of nanomaterials in applications ranging from drug delivery to imaging.^14,15^

It is also important to notice that regardless of the following of all of these guidelines and points to produce a well-designed nanomaterial with a relevant bioactive profile, these materials must still undergo approval processes that take as much time to be conducted as those for new medications, with a time frame of roughly 10 years. In regard to the direct impact on the reduction of bacterial resistance mechanisms, this time frame is once again not sustainable; however, the major advantage of nanomaterials is that they might also be applied, not directly for human application, but as a preventative tool. For example, they can be introduced onto high-risk surfaces, such as doorknobs, surgical tables and instruments, and food packaging, among others. This is readily achievable by incorporating the nanomaterials into polymeric matrixes, bypassing the extensive time frame for medication approval, the lack of specific regulations and regulatory entities for the usage of nanomaterials in a variety of fields, and allowing for their direct use in society at a faster rate, as no direct human consumption is involved.^16–19^ Regardless of their incorporation onto solid-supported devices such as polymeric matrixes, all produced nanomaterials and nanodoped materials should always be characterized and thoroughly evaluated regarding their toxicological profiles, not only toward microorganisms but also in terms of their safety for human use and exposure.

## 4 Nanoparticle Synthesis

### 4.1 Silver Nanoparticles (AgNPs)

AgNPs are one of the most extensively used and researched nanomaterials not only due to their interesting optical properties but also due to their impressive intrinsic physicochemical and bioactive properties.^20^ Lea et al.^21^ pioneered the production of silver nanoparticles in 1889, describing the synthesis of a citrate-stabilized silver colloid through a reduction process. From here, the production of silver nanoparticles has advanced significantly, with countless works reporting synthetic methods and a multitude of applications in areas as diverse as antibacterial, anticancer, nematocidal, wound healing, dentistry, and biosensing, just to name a few.^22–27^

Regardless of the widespread use of AgNPs in various fields, several studies have also reported their cytotoxic effects. These are derived from their inherent characteristics, such as their size, coating, shape, and dosage. These factors can be modulated through various mechanisms such as surface modifications or through combination with other nanomaterials.^28,29^

As AgNPs are present in materials widely available for public use, such as deodorants or antiaging creams, it is also important to consider the possible exposition routes.^29^ Studies in the literature report four main exposure routes: oral, pulmonary, dermal, or intravenous. Regardless of the exposure mechanism, their immediate toxic effects are identical, being mostly sustained by inflammation phenomena and the promotion of oxidative stress. From here, a more widespread effect can occur if the nanoparticles can breach the circulatory system, potentially causing organ-specific pathophysiological effects. Nevertheless, AgNPs are a material that shows remarkable versatility in terms of shape, size, and morphology, allowing for their application in a variety of areas and fields, provided careful consideration of their inherent characteristics is performed. Figure 2 shows an overview of several silver nanoparticles in the literature, in which it is possible to discern the various shapes and sizes that might be obtained through the manipulation of the synthetic variables.

**Figure 2:**
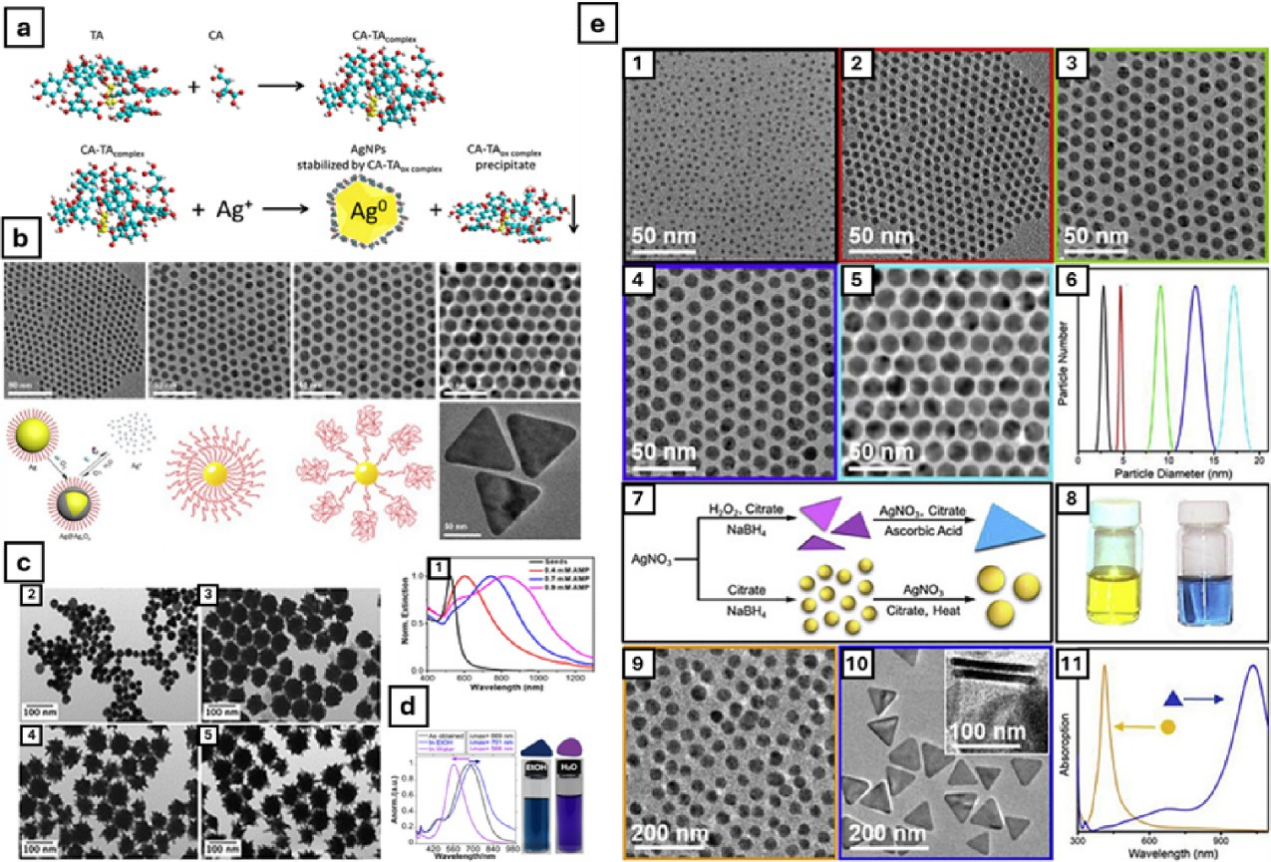
(a) Schematic overview of the role of sodium citrate (CA) and tannic acid (TA) as capping agents in the synthesis of AgNPs. Adapted with permission under a Creative Commons 4.0 from ref 30. Copyright 2017 Springer. (b) TEM imaging of various shapes of AgNPs and respective schematic overview with the presence of capping agents. Adapted with permission from ref 31. Copyright 2022 Elsevier. (c) TEM imaging of AgNPs of various size ranges and their respective lattice fringe and size histograms. Adapted with permission under a Creative Commons CC BY 3.0 from ref 32. Copyright 2014 Royal Society of Chemistry. (d) Absorbance spectra of two differently shaped AgNPs in EtOH and H_2_O and respective colorimetric profiles. Adapted with permission under a Creative Commons CC BY 4.0 from ref 33. Copyright 2019 Frontiers. (e) TEM imaging of various shapes and sizes of AgNPs with the respective size distribution and colorimetric profile for nanospheres (yellow) and nanoplates (blue). Schematic overview of the production of different shapes of nanoparticles capped with sodium citrate and absorbance spectra demonstrating the different spectral profiles for the two different nanoparticle types. Adapted with permission from ref 31. Copyright 2022 Elsevier.

#### 4.1.1 Synthetic Routes for AgNPs

As a multitude of comprehensive reviews have extensively covered the synthetic routes and mechanisms of action already reported elsewhere,^20,34^ this paper provides a concise, brief overview of the major synthetic pathways, focusing on the proposed antimicrobial mechanisms. Table 1 summarizes the major synthetic routes employed for silver nanoparticle synthesis, providing examples for each specific route.

##### 4.1.1.1 Chemical-Based Methods

Chemical methods are primarily based on the first reported synthesis of AgNPs by Lea et al.,^21^ with the vast majority involving silver reduction steps using an organic or inorganic reducing agent.^55^ Some examples of commonly used reducing agents are sodium citrate^56^ and tannic acid,^30^ which can fully coat the metallic core, stabilizing it in its zero-valence state (Figure 2a). Other examples in the literature commonly employ sodium borohydride^35^ or dimethylformamide (DMF)^56^ with a similar purpose.

Additionally, chemical synthetic routes of AgNPs can also include the presence of a capping agent to help stabilize the obtained nanoparticles and modulate their morphology.^57^ The combination and ratios of each of these components will yield nanoparticles with distinct characteristics and even different shapes, yielding nanospheres, nanorods, and nanostars, among others^31,58^ (Figure 2b,c). It is also important to notice that these different obtained shapes will yield different physicochemical, optical, and even biological activities, highlighting the influence that the synthetic routes can have on the final intended applications of each nanomaterial. An easy way to verify these alterations is not only through the colorimetric alterations, usually noticeable to the naked eye, but also through the acquisition of their respective absorption spectra, in which different shapes demonstrate red or blue shifts in their spectral profiles, corresponding to higher or lower energetic states and particle stability (Figure 2d,e). Beyond the internal synthetic components, external factors such as pH or temperature are also relevant in the synthetic environment, as they can also easily influence the shape and characteristics of the obtained nanomaterials.^56,59^

Prior to the addition of the selected silver precursor, chemical reducing agent, and capping agent in solution, the formation of AgNPs commonly follows a two-step process, consisting of a first nucleation step and a second growth step following a “bottom-up” mechanism.^60^ Other “bottom-up” chemical-based methods for the production of AgNPs are also reported in the literature, such as photochemical methods, in which the reduction of the silver precursor is achieved through irradiation,^37^ with a simultaneous occurrence of both nucleation and growth phenomena; microwave-assisted methods, in which the nucleation process occurs in response to microwave irradiation, promoting the generation of the silver nuclei;^61^ or sonochemical methods, in which cavitation effects produce a “hot-spot” promoting the generation of nuclei.^62^ All these methods have in common the reduction of the silver ions, promoting nuclei formation and later growth of the silver nanoparticles to the desired size.

##### 4.1.1.2 Electrochemical-Based Methods

Electrochemical synthesis processes for the production of AgNPs involve the reduction of silver ions at a cathode surface within an electrochemical cell. This process can have a dual interpretation, being both “top-down” or “bottom-up” depending on the selected metal precursor. In the case where a silver salt is added to the electrolyte solution in which this process is conducted, a “bottom-up” approach can be considered; however, if a bulk silver material, commonly referred to as a sacrificial anode, is placed in the reaction mixture, a “top-down” approach is considered. Regardless of the process, silver ions are released onto the medium, which can be aqueous or organic, and a nucleation process occurs, forming the nanoparticles. As this process is mediated through the application of an electrical current, the nucleation process tends to occur on the surface of the electrode. To prevent this phenomenon, surfactants and other additives are commonly added to the electrolyte solution, forming a blocking layer on the electrode itself and preventing the adsorption of the nanomaterial.^63^

Modification of parameters such as the concentration and composition of the electrolyte solutions, the cathode potentials, and the bulk materials, among others, allows for the manipulation of the size and shape of the obtained nanoparticles.^40–44^ For example, by applying organic solvents or ionic liquids as an electrolyte solution, instead of an aqueous medium, it is possible to apply higher current density, which allows for the formation of smaller particles.^64,65^ The metal ion concentration in the initial solution is another parameter that can be modified to yield different sized particles, as is also the type of surfactant used. These molecules not only contribute as a protective barrier to the electrode, preventing the occurrence of metal ion deposition onto the electrode itself, but also have an important role in the stabilization of the obtained nanoparticles in solution. Temperature is yet another important aspect to consider, as a higher energetic input onto the reactional environment might promote faster reactions, as the additional energy assists in overcoming the activation energies of the nucleation processes occurring after the reduction of the metal ions toward their nanoparticle configuration.^66^

##### 4.1.1.3 Physical-Based Methods

Physical-based synthetic methods rely on both mechanical and vapor-based methods, which intend to, through the application of energy, whether it being mechanical or electrical, physically reduce a silver precursor to a fine powder, yielding the intended nanoparticles.^34^ The ball/bead milling process is one strategy which consists briefly of the mechanical grinding of a silver precursor to produce increasingly smaller silver particles until the desired size is achieved.^67^ This process is also highly influenced by the atmosphere and temperature in which the grinding is conducted, so prior adjustment of the optimal experimental conditions is advised. Other physical methods for silver nanoparticle production also include electrical arc-discharge, laser ablation, and physical vapor deposition methodologies.^68–70^ These use direct current, pulsed laser, and a cathodic arc source, respectively, to the bulk metal, yielding the intended nanoparticles. Although effective, these methods also present some limitations such as the difficulty in precisely controlling the nanoparticle size obtained; however, methods such as electrical arc-discharge are a good low-cost choice for silver nanoparticle production through physical methods.

##### 4.1.1.4 Biological-Based Methods

Biological synthesis of AgNPs relies on the presence of naturally occurring reducing agents in plants, microorganisms, or algae. Among these, microorganisms can take advantage of obtained evolutionary mechanisms, gained when exposed to environments with high silver content, which results in the enrichment of their silver-reducing mechanisms, enabling the efficient reduction of silver ions and yielding nanoparticles as a “by-product”.^71^ Regarding the bacterial production of silver nanoparticles, a myriad of mechanisms is in place, with several enzymes playing a significant role, such as silver reductase.^72^ Algae, conversely, are more dependent on the large content of biologically active reductant agents such as carbohydrates, enzymes, vitamins, etc.^51^ The sizable presence of such compounds provides an optimal environment for silver reduction phenomena, yielding silver nanoparticles in high yield. Moreover, due to the presence of these bioactive components, bacterial and algal extracts have also been employed as capping agents for chemical-based methods for silver nanoparticle production.^73^ In a similar aspect, due to the high bioactive molecule content and their high tolerance to metal-rich environments and soils, fungi are also effective candidates for the sustainable, biobased production of AgNPs.^74,75^ A crucial factor to consider about fungi-based AgNP synthesis is the potential pathogenicity of certain fungi to humans, with several washing and further purification cycles needed to ensure the safety of the AgNPs.^34^

Plants can also be used as mediators for AgNP synthesis, with extracts from different plant parts, such as bark, flowers, or leaves, being used as reducing agents.^74,76,77^ These extracts are rich in a myriad of organic compounds such as enzymes, phenolic compounds, and terpenoid compounds, promoting the reduction of silver ions and subsequent nanoparticle formation.

Although efficient in their reducing capacity, biological methods have an associated inherent biological variability, which might make the production of homogeneous, monodisperse nanoparticles difficult. The different contents of the respective reducing agents present in each microorganism, or extract utilized, are some of the major drawbacks of this approach; however, it remains an easy, sustainable, and low-cost method for the production of AgNPs.

### 4.2 Copper Nanoparticles (CuNPs)

CuNPs, although not as extensively studied as AgNPs, are another interesting and versatile nanomaterials, with a wide range of biological applications, ranging from antimicrobial to coating agents.^78^ The two main advantages that Cu-based nanoparticles present when compared to other metallic nanoparticles are their cost-effectiveness, and the abundance of copper in nature. Due to their characteristics, CuNPs are a possible candidate for application in a variety of fields, with their antimicrobial activity already being widely studied.^79,80^ However, like AgNPs previously discussed, some toxic effects have also been reported, namely due to the mechanisms of ROS formation.^81^

A multitude of protocols can be employed to produce CuNPs, with a variety of sizes and shapes, as shown in Figure 3. Through modulations in the morphological parameters of the particles, it is possible to influence their bioactive and toxicological profiles to best fit the intended application.^82^

**Figure 3:**
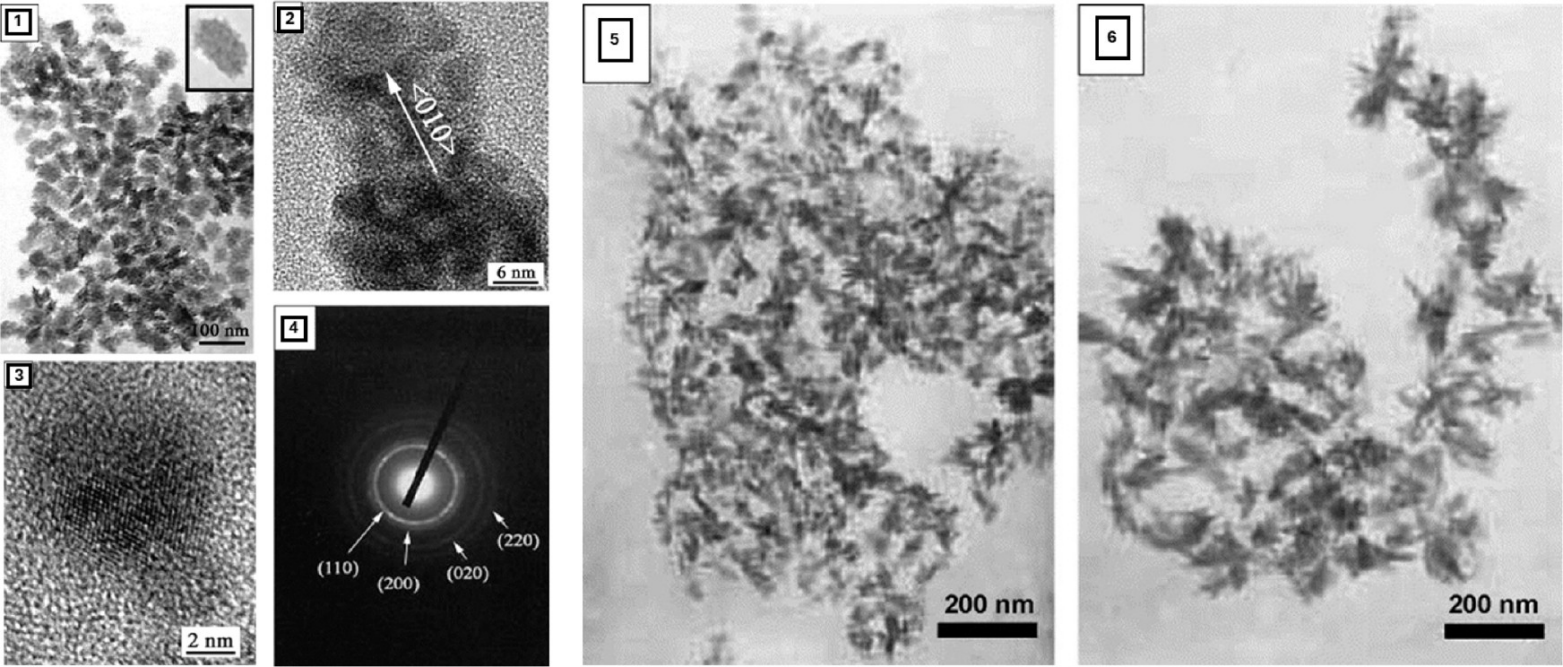
TEM imaging of several types of Cu nanoparticles. SAED patterns of a single CuNP. Adapted with permission from ref 84. Copyright 2006 Elsevier.

Similar to that already described for AgNPs, three main elements are necessary to produce CuNPs: a metal precursor to provide copper ions, a reducing agent to promote the reduction of these ions, and a surfactant that aggregates both precursor and reducing agents to produce the intended nanoparticles.^85^

Once again, four main paths can be used to produce CuNPs: chemical, electrochemical, physical, or biological. As other works extensively detail the synthetic routes for CuNP synthesis,^86^ this review will only provide a simplified version of some of the most employed methods. Table 2 summarizes the major methods used to produce CuNPs.

**Table 2:** Summary of the Various Routes for CuNP Synthesis

**Synthetic Route:** Chemical					
Method	Cu Precursor	Reducing agent	Capping/Stabilizing agent	Size (nm)	ref
Chemical Reduction	CuCl_2_	l-ascorbic acid	PVP	≃34	^83^
	CuSO_4_·5H_2_O	Ascorbic acid	Starch	≃29	^87^
	CuSO_4_·5H_2_O	Ascorbic Acid	Starch	14 to 55	^88^
	Cu(acac)_2_	1,2-hexadecanediol	Oleic acid + Oleylamine	5–25	^89^
	CuSO_4_·5H_2_O	Glucose	Oleic acid + Oleylamine	45	^90^
	CuSO_4_·5H_2_O	N_2_H_4_	Sodium metaphosphate	≃2.5	^91^
Microemulsion	CuCl_2_	NaBH_4_	AOT	≃500	^92^
	CuSO_4_·5H_2_O	NaBH_4_	SDS + Isopentanol	≃3	^93^
Microwave-assisted	CuSO_4_·5H_2_O	NaH_2_PO_2_·2H_2_O	PVP	≃10	^94^
	Copper acetylacetonate	Benzyl alcohol	Benzyl alcohol	150	^95^
Ultrasonic-assisted	CuSO_4_	N_2_H_4_	Ethylene glycol	≃108	^96^
Sonochemical	CuSO_4_·5H_2_O	NaBH_4_	CTAB	30–120	^97^
Thermal-assisted	CuSO_4_	-	-	27	^98^

**Synthetic Route:** Electrochemical				
Cu Precursor	Stabilizer	Electrode	Parameters	ref
Cu_2_O	Choline chloride and urea (DES)	Ni disk	303–333 K; 10 mC cm^–2^	^99^
CuCl_2_	Phosphate buffer 0.1 M pH 7.0	Ag/AgCl; Platinum wire	+1.1 to −0.7 V; 50 mV/s	^100^
CuCl_2_	Sargassum extract	Graphite, saturated calomel	0.1 M K_2_SO_4_ pH = 2.3 to 5 mV/s	^101^

**Synthetic Route:** Physical				
Method	Cu precursor	Size (nm)	Conditions	ref
Ball-milling	Cu(CH_3_COO)_2_	20	Low temperature	^102^
Laser Reduction	Cu(acac)_2_	100	Laser irradiation and air exposure	^103^
Laser ablation	Cu plate	4–11	Cu plate immersed in virgin coconut oil. Nd:YAG laser at 532 nm.	^104^
Thermal decomposition	Cu oxalate	40	140 °C (1 h) 245 °C (65 min)	^105^
	Cu acetylacetonate	2–4 (Cu_2_O) 6 (CuNPs)	100 °C/180 °C/220 °C 6 h	^84^

**Synthetic Route:** Biological				
Microorganism	Cu Precursor	Size (nm)	Bioactive compound	ref
Seaweed (*Ulva Lactuca*)	CuSO_4_	-	Flavonoids, glycosides, phenolic compounds, saponins, steroids, and tannins	^106^
Fungus (*Aspergillus niger*)	CuNO_3_	3–70	-	^107^
Plant (*Krameria* sp.)	CuSO_4_·5H_2_O	5–7	-	^108^

#### 4.2.1 Synthetic Routes for CuNPs

##### 4.2.1.1 Chemical-Based Methods

Chemical synthetic methods typically follow standard “bottom-up” approaches, in which the smaller units are assembled to produce a larger three-dimensional structure. A variety of chemically oriented methods can be used to produce CuNPs such as (1) chemical reduction, (2) microemulsion, (3) microwave-assisted, or (4) ionic liquid-assisted methods, to name the most employed.

Chemical reduction is, however, the major method described in the literature to produce CuNPs. In a similar procedure to what was previously described for AgNPs, this method is based on the reduction of copper ions to metallic copper using reducing agents in the presence of a stabilizing agent.^83,86,89–91^ These copper ions can originate from a copper precursor such as copper sulfate pentahydrate or copper chloride, in combination with reducing agents such as sodium borohydride, hydrazine, or ascorbic acid. On the stabilizing agents, starch or polyvinylpyrrolidone (PVP) are commonly used as well to prevent the agglomeration of the produced nanoparticles and their oxidation. Other capping agents can also be used in the synthetic process to aid in the stabilization of the metallic cores and function as morphological agents, such as ethylene diamine tetra acetic acid (EDTA).^109^ Figure 3

Factors such as pH or temperature can deeply influence the shape and size of the obtained nanoparticles, as can be verified in Figure 3, in which TEM imaging of different shaped CuNPs is presented, obtained through different synthetic approaches, being important to tightly control these parameters.^110^ By the adjustment of these parameters, it is possible to manipulate the shape, size, and overall properties of the produced nanoparticles.^111^

Microemulsion is yet another chemically based method to produce CuNPs. This method, often referred to as the reverse micelle method, is based on the creation of a water-in-oil emulsion in an aqueous environment. These micellar environments can function as catalysts for the formation of nanoparticles in the presence of a stabilizing agent and a strong reducing agent like the simple chemical reduction methods.^92,93^

Some microwave-aided techniques are also described, in which the reaction mixture is exposed to microwave radiation as an energy source to catalyze the formation of CuNPs.^94,95^ The application of energy to the reaction mixtures in the form of ultrasonic,^96^ sonochemical,^97^ thermal,^98^ or electrical^112^ energy has also been described in producing well-dispersed CuNPs.

##### 4.2.1.2 Electrochemical-Based Methods

CuNPs can be obtained by electrochemical protocols. The basis for these protocols is similar to that described in Section 4.1.1.2 for AgNPs. It involves the reduction of metal ions, in this specific case of copper ions, through the application of electric current to an electrolyte solution rather than through a traditional chemical reaction. This solution can contain either the precursor metal in a salt configuration or a bulk material, such as an electrode. From there, the metal ions present in the medium will undergo a nucleation process and subsequently form the intended nanoparticles.

Several studies in the literature point to this method as a sustainable and low-cost method to produce nanoparticles, allowing for a precise control of their morphological characteristics such as size and shape. Variations in the conditions of the synthetic procedures can easily modulate the obtained nanoparticles,^83,113^ for example, through the use of deep eutectic solvents instead of the traditional aqueous electrolyte solutions^99^ or even through the use of plant extracts.^101^ This technique also allows for the coating of other surfaces through the deposition of copper nanoparticles onto their surface.^100^

##### 4.2.1.3 Physical-Based Methods

Unlike chemical methods, which employ “bottom-up” approaches, physical-based methods employ a “top-down” approach for the synthesis of CuNPs. These methods are based on the decomposition of a bulk material to produce the intended nanoparticles. Several techniques might be applied to this end, some involving brute force, as is the case with ball milling techniques.^102^

Other strategies involving a different energetic application, for example, through laser reduction, in which the energy is directed to the bulk material to ease its decomposition, are also reported in the literature, forming the intended nanomaterials.^103^ These techniques can be applied in a variety of environments, for example, in the irradiation of a Cu plate submerged in virgin coconut oil, yielding CuNPs with diameters ranging from 4 to 11 nm.^104^ Thermal energy has also been proven to produce the decomposition of Cu precursor materials, such as Cu oxalate^105^ or Cu acetylacetonate,^84^ to produce CuNPs and copper oxide nanoparticles (CuO).

One main advantage of these methods is the purity of the obtained materials, as no other reagents are necessary and the nanoparticles are produced from a pure bulk material. However, all these consume high levels of energy and commonly require the use of expensive devices to allow for nanoparticle formation.^78^

##### 4.2.1.4 Biological-Based Methods

CuNPs might also be produced through biological methods, which use biological agents, such as fungi, plants, or bacteria as reducing agents, which are biocompatible and nontoxic by nature.^106–108,114^ These protocols use extracts from plants or microorganisms, as these have elevated levels of phenolic, terpenoid, and flavonoid compounds, as well as enzymes, which can function as stabilizers, capping agents, and reducing agents. These are methods of high interest not only due to their associated low costs but also due to the ease with which the process can be conducted prior to extract obtention. Regardless of their sustainable profile, it is important to consider, however, that the use of biological agents as reducing agents can introduce increased variability into the obtained nanomaterials. This variability arises due to the inherent biological variability of the extracts, as previously described in Section 4.1.1.4 of this review. Additional disadvantages concerning the microorganism-based routes include the handling of microorganisms in an aseptic environment, increasing production costs and the time required for these processes.^78^

### 4.3 Silver–Copper Nanoparticles (AgCu NPs)

#### 4.3.1 Synthesis Routes for AgCu NPs

A possible method to mitigate the difficulties associated with any specific type of monometallic nanoparticle is through the combination of two or more monometallic counterparts, producing a polymetallic material. Among these, some of the most synthesized nanoparticles are bimetallic nanoparticles. These materials derive synergistic interactions between their individual elements, producing a combined material with improved inherent properties, for example, presenting better catalytic and photocatalytic properties, modulation of localized surface plasmon resonance (LSPR) to best achieve an intended goal, or even increasing the magnetic properties of the materials.^115^

The combination of Ag and Cu to produce hybrid AgCu alloys is a promising strategy to overcome some hindrances presented by the monometallic materials themselves, whether it be the rapid oxidation phenomenon experienced by Cu or electromigration phenomena present in Ag,^116^ taking advantage of their benefits. More synergistic mechanisms involving both metals have been demonstrated in the literature, for example, related to selective CO_2_ reduction mechanisms, potentiated by the presence of Ag metal ions in a Cu-rich environment, through the production of AgCu foam catalysts as demonstrated by Kottakkat et al.,^117^ or for the production of a conductive ink, suitable for application in printed electronic devices, through the synthesis of AgCu core–shell nanoparticles as described by Pajor-Świerzy et al.^118^ From these examples, it is possible to understand the scientific and industrial potential that the stabilization of these two metals in an alloy configuration can have. Despite this interest and the potential these materials present, few studies have been reported in the literature. The theoretical properties of these materials, such as the energetic states between the various nanoparticle configurations, as can seen in Figure 4a, have been evaluated through molecular dynamics and computational studies.^119–128^ However, in more recent years, these studies have evolved to the actual production and evaluation of such materials.

**Figure 4:**
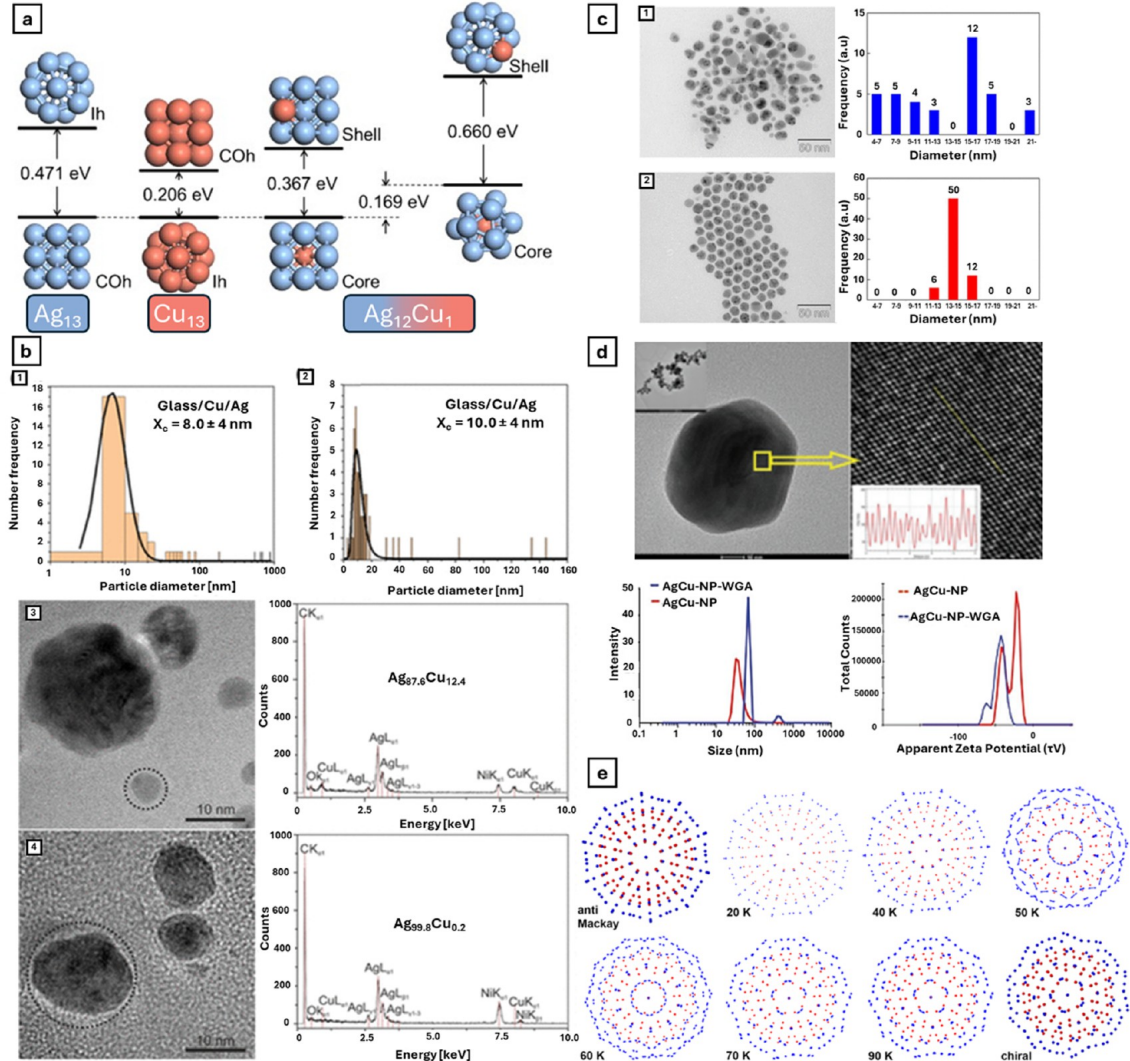
(a) Energy diagrams demonstrating the difference between energetic states of various Ag (blue) and Cu (red) structures. Reprinted with permission from ref 121. Copyright 2011 Elsevier. (b) TEM imaging of various Ag/Cu/glass nanocomposites, respective size distribution histograms, and EDX spectra. Reprinted with permission from ref 120. Copyright 2021 American Chemical Society. (c) TEM images and size distribution histograms of AgCu NPs. Reprinted with permission from ref 129. Copyright 2021 American Chemical Society. (d) TEM characterization of AgCu NPs with the respective size distribution histogram, lattice fringes, and hydrodynamic diameter characterization performed by Dynamic Light Scattering spectroscopy. Reprinted from ref 130. Copyright 2022 John Wiley and Sons. (e) Schematic representation of Ag (blue) and Cu (red) distribution in nanostructures as a function of temperature. Adapted with permission under a Creative Commons CC BY 4.0 from ref 119. Copyright 2020 Springer Nature.

Few studies in the literature have reported the production of AgCu bimetallic alloys. The production of these materials can have some challenges associated. Due to the difference in lattice constants presented by each material, 0.361 nm and 0.409 nm, respectively, for Cu and Ag, the stabilization of the metals in a single alloy can be difficult.^131^ Furthermore, both metals also have different reduction potentials, + 0.80 *E*_o_ (V vs SHE) for Ag­(I)/Ag(0) and +0.34 *E*_o_ (V vs SHE) for Cu­(II)/Cu(0),^132^ making it difficult to control the simultaneous reduction of both metals to form a homogeneous alloy. Additionally, copper is also known to be of poor stability in aqueous media, further increasing the difficulty to synthesize and stabilize a AgCu alloy. Despite these challenges, studies on the production of AgCu hybrid nanoparticles in the literature report a variety of methods for their production, with different shapes and size distributions, as seen in Figure 4b–d. Table 3 summarizes these procedures. These can be divided into four main categories: chemical-, electrochemical-, physical-, or biological-based methods.

**Table 3:** Summary of the Methods for the Synthesis of Bimetallic AgCu NPs

**Synthetic Route:** Chemical						
Method	Ag Precursor	Cu Precursor	Reducing agent	Capping agent	Size (nm)	ref
Chemical Reduction	AgNO_3_	Copper acetate	Hydrazine and sodium citrate	Mercaptopropionic acid	4–32	^133^
	AgNO_3_	Copper acetate	-	PVP	ca. 180	^134^
	Silver citrate	Copper acetate	NaBH_4_	PVP	45–50	^135^
	AgNO_3_	Copper acetate	Cysteine	Sodium citrate	-	^136^
	AgNO_3_	Copper nitrate	Cysteine	-	ca. 4.18	^137^
	[Ag (NH_2_C_12_H_25_)_2_] NO_3_	[Cu(PPh_3_)_2_(bea)] [Cu(PPh_3_)_3_(Hphta)]	Oleylamine	1-octadecene	ca. 13–14	^129^
	AgNO_3_	Copper acetate	Hydrazine	-	-	^130^
	AgNO_3_	Copper chloride	NaBH_4_	TPPB	1.69	^138^
	AgNO_3_	Copper sulfate	l-ascorbic acid	PVP	40	^139^
	AgNO_3_	Cu(acac)_2_	Oleylamine	-	13.9	^140^
	AgNO_3_	Copper sulfate	Sodium citrate	-	50–150	^141^
	AgNO_3_	Copper acetate	Oleylamine and 1-octadecanol	Oleic acid	15	^116^
Deposition	Commercially available AgNPs	Copper nitrate	-	PVA and PVP	15–22	^142^
	Aqueous AgNPs dispersion	Copper nitrate	-	PVA and PVP	3–20	^143^
	Aqueous dispersion AgNPs	Copper nitrate	-	PVA and PVP	6–15	^144^
	Aqueous dispersion of AgNPs	Copper nitrate	-	PVA and PVP	1–22	^145^
Microwave assisted	AgNO_3_	Copper nitrate	Ascorbic acid	Starch	32–55	^131^
	AgNO_3_	Copper sulfate	Tannic acid	-	13–156	^146^
Microemulsion	AgNO_3_	Cu(AOT)_2_	Hydrazine	-	3	^147^
Galvanic Displacement	AgNO_3_	Cu NPs commercially available	-	-	100–200?	^148^

**Synthetic Route:** Electrochemical					
Ag Precursor	Cu Precursor	Stabilizer	Electrode	Parameters	ref
AgNO_3_	CuSO_4_	Hydrazine	Carbon rods	0.01 A/s	^149^
AgNO_3_	CuSO_4_	-	Foams	25 s pulse of –0.9 V	^150^

**Synthetic Route:** Physical					
Method	Ag precursor	Cu precursor	Size	Conditions	ref
Deposition	Ag layer	Cu layer	-	Annealing of the layers at various temperatures	^151^
	Ag layer	Cu layer	-	Eutetic ratio of 28 wt % Cu	^152^
	AgCu square plate		-	Laser ablation with a KrF laser	^153^
Sputtering	Ag wire	Cu target	-	6 cm of distance to the target and 6 rpm	^154^
	Pure Ag target	Pure Cu target	-	High vacuum cosputtering system	^155^
	Pure Ag target	Pure Cu target	-	Deposition temperature of 20 °C, base pressure of 1.0 × 10^–2^ mbar, Ar flow and sputtering power of 40 W	^156^
Hydrogen-reduction-assisted Ultrasonic Spray Pyrolysis	AgNO_3_	(CuNO_3_)_2_·3H_2_O	-	Percursor solution atomized and the reduction of aerosol droplets occurred at 800 °C	^157^
Pulsed plasma in liquid	Electrode	Electrode	-	5000 vibrations per minute	^158^
Pulsed Layer Ablation	CuAg bilayer films		-	Film thickness of ca. 80 nm	^120^

**Synthetic Route:** Biologic					
Microorganism	Ag Precursor	Cu precursor	Size	Bioactive compound	ref
*Achras sapota* Linn	AgNO_3_	Cu(NO_3_)_2_·3H_2_O	20–40 nm	Reducing sugars, proteins, ascorbic acid, phenolics and carotenoids	^159^
*Aegle marmelos* and *Citrus limetta*	AgNO_3_	CuSO_4_	5–7	Full extract	^160^
*Phoenix dactylifera*	AgNO_3_	Cu(NO_3_)_2_·3H_2_O	-	Phenolic compound	^161^
*Kigelia Africana*	AgNO_3_	CuCl_2_·2H_2_O	ca. 10 nm	Full extracts	^162^
*Aspergillus terreus*	AgNO_3_	CuNO_3_	20–22	Full fungal biomass	^163^

##### 4.3.1.1 Chemical-Based Methods

Most methods described in the literature for the synthesis of AgCu NPs typically follow chemically based methods in a “bottom-up” approach, relying additionally on a thermal part to ease the reduction process of both metal precursors and to help stabilize the obtained nanomaterials. Temperature is a crucial factor to consider with regard to the production of AgCu NPs, as different temperatures can yield different distributions of both monometallic counterparts in the final nanoparticle, as seen in Figure 4e.^119^ To perform the metal reduction process, a strong reducing agent is needed, to reduce both metal precursors to their zerovalent state.^133^ Additionally, capping agents such as polyvinylpyrrolidone (PVP), polyethylene glycol (PEG), or cetyltrimethylammonium bromide (CTAB) might also be added to stabilize the obtained nanoparticles and prevent aggregation and oxidation phenomena.^134,135^

The key issues reported in the synthesis of AgCu NPs are related to their low stability, high air sensitivity, and the high reactivity of copper. These oftentimes originate the presence of copper oxides in the final product.^136^ To minimize these issues, synthetic routes are commonly conducted under an inert atmosphere. However, through the regulation of all these parameters, precise control over the characteristics of the produced particles, such as their size, shape, and metal composition percentages, can be achieved.

One of the earliest reports on AgCu nanoparticles underlines the importance of reagent choice, as different combinations might yield materials with different properties. Various metal ratios and solvent iterations were able to produce a wide range of bimetallic nanoparticles, with various configurations ranging from a pure AgCu alloy to Cu core-Ag shell and Ag core-Cu shell nanoparticles, confirmed through different LSPR bands and transmission electron microscopy (TEM) analysis. These nanoparticles ranged in size from 35 to 40 nm. The results obtained through reagent variations clearly illustrate the adaptable profile of these synthetic routes, producing stable and versatile nanoparticles. Other more complex systems have also been reported through the addition of other elements to the reaction mixture. Taner et al.^136^ report the introduction of cysteine into the reaction mixture as an antioxidant agent to prevent the oxidation of metallic Cu to the previous state of Cu^2+^. Zhang et al.^137^ have also introduced cysteine as a stabilizing agent; however, in a different approach, to produce an AgCu nanocluster hydrogel. Interestingly, a green-chemistry-directed approach was performed by using an ultraviolet (UV) photoreduction one-pot method.

Although hybrid AgCu NPs can be produced using metal salts as precursors, other silver and copper complexes can also be used for this function. Vykoukal et al.^129^ report the production of AgCu nanoalloys using as a silver precursor [Ag­(NH_2_C_12_H_25_)_2_]­NO_3_ and as copper precursors [Cu­(PPh_3_)_2_(bea)] and [Cu­(PPh_3_)_3_(Hphta)]. Interestingly, through the differentiation of these two copper precursors, two vastly different shapes of nanomaterials were obtained. By using [Cu­(PPh_3_)_2_(bea)], Janus-type nanomaterials were obtained with diameters of 15–17 nm, contrasting with the Ag/Cu alloy mixture obtained with the second precursor, yielding spherical, more homogeneous, and monodispersed 13–15 nm diameter nanoparticles. Once again, the type of precursor used highly influenced the obtained nanomaterials about shape, size, and nanoparticle distribution.

The choice of the reducing agent is yet another crucial aspect of the hybrid AgCu NP synthesis. Out of all of the reported studies, hydrazine is the most often reported to this end. Hydrazine is a strong reducing agent, which is quite effective in reducing transition metal oxides.^164^ Nonetheless, it is quite hazardous and expensive, hindering large scale-ups of the synthetic process. Still, various authors have reported in the literature the use of hydrazine as a reducing agent for the synthesis of hybrid AgCu NPs^130,133,136^ due to its strong nature. Nonetheless, others such as sodium borohydride,^138^ ascorbic acid,^139^ oleylamine,^140^ or sodium citrate^141^ have also been reported. One point in common in the majority of reducing agents, such as those reported, is their hazardous and toxic nature. Greener alternatives, such as 1-octadecanol, have also been reported in the literature as mild, less toxic reagents that can also produce monodisperse nanoparticles, as reported by Dou et al.^116^ This study proposed the synthesis of alloy AgCu NPs with varying Ag:Cu ratios. The authors found that an equal proportion of metals was ideal to produce monodisperse spherical nanoparticles with an average diameter of ca. 11 nm.

The obtained AgCu nanoparticles can also become part of more complex systems through other functionalization of metal layers in the obtained alloys, allowing them to produce increasingly complex systems. Tang et al.^139^ report the production of Janus-type AgCu NPs, which were additionally covered by layers of Pt and Pd, producing quadrometallic nanoparticles.

##### 4.3.1.2 Deposition-Based Methods

Premade or commercially available monometallic nanoparticles can be used as a basis for the production of bimetallic nanoparticles. This type of synthesis often focuses on the deposition of metal ions onto an already existent core nanoparticle, forming a core–shell structure instead of a mixed alloy. To this end, commercially available AgNPs were used as a basis to produce silver core-copper shell nanoparticles.^142,143^ To these core silver NPs, different copper precursors were added, varying their concentrations in the initial mixture to best optimize the Ag:Cu ratio present in each material. As an example, Zhou et al.^144^ report one such process, in which commercially available AgNPs, stabilized with a mixture of PVP and PVA, serve as a basis for the deposition of Cu ions. This process was also conducted with iron metal ions, proving the versatility that it presents. Stabilized AgNPs can catalyze the metal ions added to the reaction mixture, under high temperatures, causing their direct deposition on the surface of the particle, yielding spherical nanoparticles with diameters ranging from 15 to 50 nm. In another example, Tao et al.^145^ followed a similar approach, by depositing copper nitrate onto PVA–PVP stabilized AgNPs at 60 °C. Once again, spherical nanoparticles were produced. Interestingly, the size of the nanoparticles was able to be modulated according to the reaction time, with the hybrid AgCu occurring after only 10 min of reaction. All synthetic mechanisms that depict this strategy can be accompanied by the acquisition of UV–vis spectra, as the initial silver nanoparticles present a well-defined plasmonic resonance band, which is modulated with the reduction and subsequent deposition of copper ions on the surface of the particle, producing the hybrid nanomaterial. Differences in the absorption and light scattering of the material might cause a shift or decrease in the intensity of the original AgNPs’ UV–vis plasmonic resonance band, as verified in the above-mentioned studies.

##### 4.3.1.3 Microwave-Assisted Methods

Microwave-assisted techniques have also been reported as a greener alternative for the chemical production of hybrid bimetallic AgCu NPs. Valodkar et al.^131^ reported the first production of stable spherical nanoparticles using microwave-assisted methods. Several Ag:Cu ratios have been evaluated, producing nanoparticles with sizes ranging from ca. 32 to 52 nm of diameter. To the metal precursors, silver and copper nitrate, were added starch and ascorbic acid as stabilizing and reducing agents, respectively. A particular advantage of this method is the one-pot type of synthetic route as well as the quick time, as these nanoparticles were obtained after only 90 s in a common microwave oven at maximum power. Another example has been reported by Reyes-Blas et al.^165^ in a similar one-pot approach, using as metal precursors silver nitrate and copper acetate, in the presence of ethylene glycol as a solvent and sodium chloride, PVP, and NaOH, for 2 min at a temperature of 175 °C. Once again, stable nanoparticles were obtained, although these parameters yielded much smaller nanoparticles, with an average diameter of ca. 12 nm and a quasi-spherical irregular shape. An Ag:Cu ratio of 2:1.5 was determined to be optimal for this synthetic route; however, it might be modulated to produce nanoparticles with varied sizes and to adjust the shape of the obtained nanoparticles. In a more recent study, Dlugosz et al.^146^ have also used a microwave-aided technique to produce hybrid AgCu NPs, using once again as metal precursors silver nitrate and copper sulfate, in a flow system run in a microwave oven. A two-step approach was proposed, yielding nanoparticles with sizes ranging from ca. 27 to 96 nm, once again dependent on the Ag:Cu ratio of the initial reaction mixture. All these processes have in common the fast time with which the nanoparticles are produced and the lack of more toxic components in the reaction mixtures, allowing for a fast, high-throughput synthetic route for this type of nanomaterial.

##### 4.3.1.4 Other methods

Other chemical-based techniques have also been reported, although in a smaller number, such as a microemulsion method reported by Tanori et al.^147^ The microemulsion method combines two immiscible aqueous/oil phases and through coalescence phenomena catalyzed by a precipitating agent produces the intended nanoparticles. This process yielded spherical stable nanoparticles with an average diameter of about 3 nm.

Galvanic displacement is yet another technique reported for the synthesis of AgCu hybrid materials and may be associated with other techniques, as reported by Du et al.,^148^ in which the production of copper-core silver-shell nanomaterials is described. The materials reported in this study follow a deposition route assisted with ultrasounds and a combined galvanic displacement technique. Kim et al.,^140^ have also reported the synthesis of AgCu NPs, which were produced using Cu­(acac)_2_ as a copper precursor. This process was based first on the production of Cu (0) nanoclusters, at a high temperature (220 °C), followed by a decrease in temperature to continue the Ag addition to the material through a galvanic displacement technique. This technique has also been reported to produce other materials such as foams for applications ranging from battery components^166^ to CO_2_ ^117^ or biomass reductors.^150^

##### 4.3.1.5 Electrochemical-Based Methods

Electrochemical methods are yet another strategy that has been reported for the synthesis of these materials. Abdul Salam et al.^149^ have reported the production of AgCu nanoalloys following an electrochemical-based method. While the study reports the production of spherical nanoparticles with modifiable size and shape, it also acknowledges an inherent challenge related to electrochemical techniques, specifically concerning method reproducibility. Other examples described in the literature for the production of bimetallic AgCu materials are through the electrodeposition of silver ions onto a copper foam, forming a bimetallic coated material^150^ or by the production of both intermixed, and phase-separated AgCu particles deposited onto carbon paper electrodes.^167^ Despite the various studies demonstrating that it is possible to produce several configurations of AgCu bimetallic nanoparticles through electrochemical methods, some protocol parameters may prove challenging to fully regulate and control, and even minor adjustments could affect nanoparticle morphology, resulting in a higher method variability than those of other methods here reported. Despite this, the approach shows promise, albeit requiring comprehensive process optimization.

##### 4.3.1.6 Physical-Based Methods

Most of the reported articles on physical-based methods for AgCuNPs production are based on deposition techniques.^168^ These can be based on the deposition of silver atoms on a copper grid, following a specific crystallographic plane, Cu (111), which yields a triangular pattern verified in the Ag layer, or the deposition of both Ag and Cu atoms on a light-emitting diode (LED) substrate, through a physical vapor deposition (PVD) technique, i.e., e-beam evaporation, resulting in the production of a multilayer electrode,^151^ or even using KCl crystals as substrates for the production of AgCu nanoparticles.^152^ A different PVD approach can also be used, through a pulsed layer deposition (PLD) protocol, to deposit an AgCu alloy onto an Al_2_O_3_ target.^153^

Sputtering methods have also been reported as strategies to produce AgCu NPs. These can be based on the deposition of only one metallic element, such as the deposition of silver atoms onto a Cu target,^154^ or through cosputtering methods, in which both metal ions are deposited onto a glass surface^155^ or in a metallic target.^156^ Other methods to produce a variety of AgCu NPs such as hydrogen-reduction-assisted Ultrasonic Spray Pyrolysis (USP),^157^ Pulsed Plasma in liquid (PPL),^158^ or Pulsed Layer Ablation (PLA) in liquids^120^ have also been reported.

##### 4.3.1.7 Biological-Based Methods

Although few, some studies on the production of bimetallic AgCu NPs have been reported in the literature. These are based mostly on the use of plant extracts, which are rich in phenolic components and ascorbic acid, as reducing agents for the synthetic route. Extracts from leaves or fruit peels of plants as varied as *Achras sapota* Linn.,^159^ *Aegle marmelos* and *Citrus limetta*,^160^ *Phoenix dactylifera*,^161^ or *Kigelia africana*.^162^ Fungal biomass has also been employed as a stabilizing and reducing agent provider, using additional microwave assistance to promote the synthesis of AgCu nanoparticles.^163^

## 5 Nanoparticle Incorporation into Polymers

The integration of nanoparticles into polymeric matrices represents a promising research area. Due to the easily manipulable nature of nanoparticles, their introduction onto solid-supported matrixes allows for the production of smart, versatile materials that can be fine-tuned and optimized for specific applications such as the production of a material with antimicrobial activity, as seen in Figure 5a.^169^ AgNPs can be clearly seen incorporated onto a cellulose nanofibril polymeric matrix. Upon testing these films against Gram-positive and Gram-negative bacteria, a clear inhibition zone is detected, indicating broad-spectrum antimicrobial activity. Additionally, the incorporation of nanoparticles onto a solid-supported matrix might also help in the stabilization of the particles themselves, further improving the characteristics of the materials.^170^

**Figure 5:**
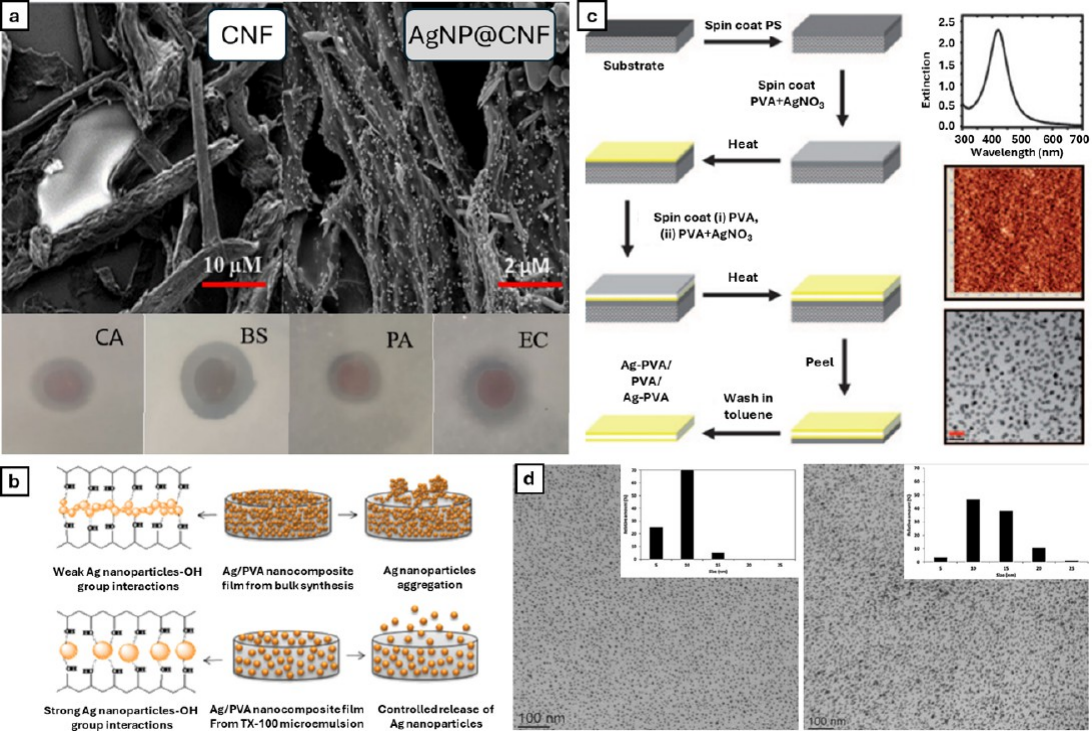
(a) SEM imaging of cellulose nanofibrils doped with AgNPs and inhibitory behavior of the nanoparticle-doped polymer against *C. albicans* (CA), *B. subtilis* (BS), *P. aeruginosa* (PA), and *E. coli* (EC). Adapted with permission under a Creative Commons CC-BY 4.0 from ref 169. Copyright 2020 Springer Nature. (b) Schematic representation of the incorporation of nanoparticles onto a polymeric matrix and the subsequent release of the particles from the matrix. Reprinted with permission from ref 171. Copyright 2017 Elsevier. (c) Schematic overview of the process of production of spin-coated AgNPs containing PVA films and respective AFM topographic profile and TEM imaging. Reprinted with permission from ref 172. Copyright 2010 John Wiley and Sons. (d) TEM imaging and respective size distribution histogram of silver nanoparticle-doped polymers. Reprinted with permission from ref 173. Copyright 2015 Elsevier.

### 5.1 Solvent Casting

The solvent casting technique is one of the most reported in the literature due to the ease with which it can be conducted. It is based on the dissolution of the polymeric material in an appropriate solvent, stabilizing the three-dimensional polymeric mesh. This liquid stabilization allows for the entrapment of additional compounds or, in the specific case of this review, nanoparticles in the matrix before its consolidation onto a solid dried film/membrane.^174^ A subsequent controlled evaporation of the solvent, whether at room temperature or in a temperature-dependent process, allows for the consolidation of the matrix onto a solid-supported material.

Its widespread use is also related to the lack of need for expensive materials, only needing commonplace solvents, be it water or any kind of organic solvent that can be easily accessible in a common laboratory. However, this same advantage can also be a hindrance on the scaling up of these same processes, as it would require the evaporation of large quantities of solvents, in a process which may not be ideal for an industrial application. Regardless, for more hands-on applications and research development, it remains one of the most employed techniques.

An important aspect when considering the drying techniques employed for the obtention of films is temperature dependence. For some organic solvents, simple room-temperature slow evaporation may be enough to produce the polymeric matrix; however, some procedures require temperature-dependent steps, whether to promote polymerization onto a tridimensional mesh or to dry the solvent, allowing for the obtention of a uniform polymeric material.

To produce nanoparticle-doped polymeric matrixes using solvent casting methodologies, two major pathways can be taken: *in situ* and *ex situ*.^175^ *In situ* production routes focus on the simultaneous production of the nanoparticles in the polymeric matrix, whereas *ex situ* techniques focus on the previous production of the nanoparticles before their introduction onto the polymeric matrix.

Considering *in situ* methodologies, these commonly include the introduction of the metal precursor, such as silver nitrate or copper chloride, for AgNPs and CuNPs respectively, into the polymeric matrix before its stabilization in a liquid environment.^175,176^ The incorporation of the precursor materials is reported to produce more well-dispersed and homogeneous polymers than through *ex situ* methodologies, in which aggregation phenomena of the particles on the matrix are more likely to occur.^177^ However, this strategy might also result in the production of less homogeneous particles, and further functionalization might not be feasible, limiting the applicability of the produced material.

Regardless, the introduction of more components into the polymeric matrix might assist in the production of more distinct materials. These additives might be reducing agents, which will assist in the reduction of the precursor materials, facilitating nanoparticle production, but can also perform as cross-linking agents, assisting and improving the mechanical characteristics of the produced polymers. One such example is reported by Kukushkina et al.,^178^ in which tannic acid and glutaraldehyde were incorporated into the chitosan matrix along with the silver nitrate precursor. These two additives acted not only as reducing agents but also as cross-linking agents, further improving the characteristics of the produced films.

One of the major advantages of this method is that it can be applied to a wide variety of polymeric matrices, from petrochemical-derived ones, such as PVA (Figure 5b),^171,178^ polyvinylpyrrolidone (PVP),^179^ or poly­(ether imide) (PEI),^180^ to biocompatible matrices such as cellulose^173^ or starch (Figure 5d).

In contrast, *ex situ* routes focus on a two-step application in which the nanoparticles are produced in a separate synthetic procedure and only then stabilized and incorporated into the polymeric matrix. The separation of the production of the materials into two distinct phases: the synthesis of the nanoparticles and the subsequent incorporation onto a polymeric matrix, ensures the optimization of both components in their respective protocols.

Additionally, it is easier to define and optimize the production of nanomaterials, tailoring them toward a specific environment without their entrapment in a polymeric matrix. This technique can be applied to a variety of polymeric matrices, such as pure PVA,^181^ blended PVA mixtures,^182,183^ agar,^184^ poly­(lactic acid),^185^ starch,^186^ polyurethane,^187^ and poly­(ethylene oxide).^188^ Copper nanoparticles have also been incorporated into polymeric matrices using similar approaches, for example, incorporating them into chitosan^189^ or even pyrrolidone ones.^190^

Another *ex situ* approach that can be taken is the introduction of a polymeric capping agent during the synthesis protocol of the nanoparticles, such as PVA that is then subjected to a temperature-dependent^191^ or ultrasound-mediated^192^ solvent casting mechanism, producing the nanoparticle-doped films, without the need for an external polymeric source.

### 5.2 Spin Coating

Spin-coating techniques are yet another widely reported technique for the production of polymeric materials.^172^ This technique is based on the deposition of a polymeric precursor solution/suspension onto a high-speed rotating disc, followed by solvent evaporation, which results in the production of a membrane/surface coating.^193^

This process is typically composed of four different stages: deposition, spin-up, spin-off, and evaporation.^194,195^ Generally, the first step comprises the deposition of the solution, which might contain precursor moieties, nanoparticles, or other additives, onto a spinning substrate. Several types of substrates can be applied, including silicon wafers, glass substrates.^196^ and polystyrene layers.^172^ Protocol iterations might also be performed to adjust the protocol to the specific matrices employed, for example, through static or dynamic depositions,^197^ which will influence the obtained polymers in terms of thickness and homogeneity.

Similar to what was described for the solvent casting methods, both *in situ* and *ex situ* techniques can be applied. *Ex situ* methods allow for the incorporation of previously synthesized nanoparticles onto a polymeric matrix. Several examples of this technique are reported in the literature using diverse polymeric matrices, for example, poly­(methyl methacrylate) (PMMA),^198^ cellulose/polyaniline,^199^ polyacrylonitrile,^200^ polyurethane,^187^ or PVA.^201–203^ The most important step for the stabilization of these particles onto a polymeric matrix is the compatibilization between the solubilities of the particles and the polymer. As the particles are stabilized in the polymeric suspension, the spin-coating cycles can be adjusted to the specific conditions of the intended polymer and allow for the production of films with the desired characteristics.

Additionally, through this method, there is a possibility of introducing distinct types of nanoparticles and other metallic components in the polymeric matrix suspension, allowing them to produce more complex nanocomposite materials in a simple approach.^204^

As also previously described, *in situ* methods are focused on the stabilization of a metallic precursor onto the polymeric matrix and subsequent spin coating onto an appropriate surface. For example, Hariprasad et al.^172^ describe the production of a nanocomposite polymeric material in which AgNPs are formed inside a polymeric PVA matrix (Figure 5c). These particles were produced only prior to the incorporation of the precursor, in this case AgNO_3_, onto the PVA matrix, followed by a spin coating cycle. The obtained matrix was then subjected to heating cycles, allowing for the formation of small monodispersed AgNPs incorporated in the matrix. As the precursor is previously incorporated into the matrix, there is good dispersion of the particles throughout the matrix. Similar approaches have also been reported in the literature with different polymeric matrices, such as the study reported by Lyutakov et al.,^205^ in which the silver nitrate precursor was stabilized onto a solubilized PMMA matrix in the presence of *N*-methyl pyrrolidone as a cosolvent and reducing agent. This suspension was then subjected to a spin coating cycle, yielding a homogeneous yellow polymer. Once again, these polymers were also subjected to high-temperature cycles to further potentiate the reduction of the precursor material and form embedded nanoparticles in the matrix.

Production of bimetallic-doped materials is also possible with this approach.^206^ Some studies have reported the common incorporation of silver nitrate as a silver precursor along with copper nitrate in the polymeric suspension prior to the application of spin-coating cycles. These studies report that the presence of a bimetallic component in the matrices improves the stability of the films and some of their mechanical and physical characteristics such as their water solubility and permeability.

### 5.3 Electrospinning

Whereas the spin-coating method is based on centrifugal force to force the dispersion of the polymeric matrix, along with any compounds that are to be doped into it, onto a collector, electrospinning methods use the application of an applied high-voltage electric field that disperses a polymeric matrix while also allowing for the melting of certain polymers and their physical manipulation. This manipulation occurs through spray-stretching phenomena, mediated by static electricity.^207^ The obtained fiber mesh can be modulated through parameters such as surface tension, flow rate, applied voltage, and polymer composition.^208^ As this technique allows for the formation of nanofibrous membranes, it becomes a versatile alternative to produce nanoparticle-doped materials with a well-dispersed presence of particles.

This is yet another method described in the literature for the incorporation of nanoparticles onto a polymeric matrix. Once again, this technique can be applied to a variety of polymeric matrices, both single and blended, and allows for the incorporation of any type of nanoparticles, as long as these are able to stabilize with the matrix, prior to the electrospinning procedure. The production of blended matrices is also another possibility. Studies report the production of matrices as varied as chitosan/polylactic acid,^209^ polylactic acid/curcumin,^210^ polyvinylidene fluoride (PVDF),^211^ PVA,^212^ polyurethane,^213^ PVP,^214^ polycaprolactone,^215,216^ chitosan,^217^ polyacrylonitrile,^218,219^ and poly­(ethylene oxide),^220^ just to name a few.

### 5.4 Other Methods

Several other techniques have been reported in the literature for the incorporation of nanoparticles onto polymeric matrixes. Although yielding well-dispersed, homogeneous polymers, these are reported in a fewer number in the literature and oftentimes produce coatings instead of independent films/membranes, while using more expensive and more specialized equipment. Regardless, a brief list of these techniques is still presented in this section. These can be related to plasma polymerization, in which, briefly, the polymeric matrix suffers from an abrupt energy input, from radiofrequency or microwave application, activating the monomeric moieties through the formation of free radicals on the polymeric surface and triggering a subsequent polymerization of the material.^221^ Some studies have reported the successful incorporation of both silver and copper nanoparticles through this technique in polymers such as polymethylsiloxane,^222^ plasma polymer films,^223^ poly­(allyl alcohol),^224^ or even Cu-plasma polymerized fluorocarbon matrices.^225^ Other techniques such as electrodeposition,^226^ sputtering,^227^ and electropolymerization^228,229^ have also been reported.

## 6 Antimicrobial Mechanisms of Action

The antimicrobial activity of metallic nanoparticles is widely reported in the literature.^230–233^ Although the specific mechanisms of action are not yet fully understood, it is a widely accepted hypothesis that this activity is not derived from only one antimicrobial mechanism but from a combination of factors and interferences that originate from the interactions of the nanoparticles with the microorganisms (Figure 6a). The influence of each factor varies depending on the specific microorganism’s species or type. However, the overall effect arises from a combination of factors that work simultaneously to produce a significant biological response, leading to outcomes such as cell death, growth inhibition or apoptotic behavior. Biological variability and environments also play a key role in the antimicrobial activity of nanoparticles. For example, inhibiting a microorganism that has already infected another body is a different task than inhibiting the same microorganism prior to infection. Different biological environments might also modulate the response that the microorganism presents toward a specific antimicrobial agent, such as nanoparticles.

**Figure 6:**
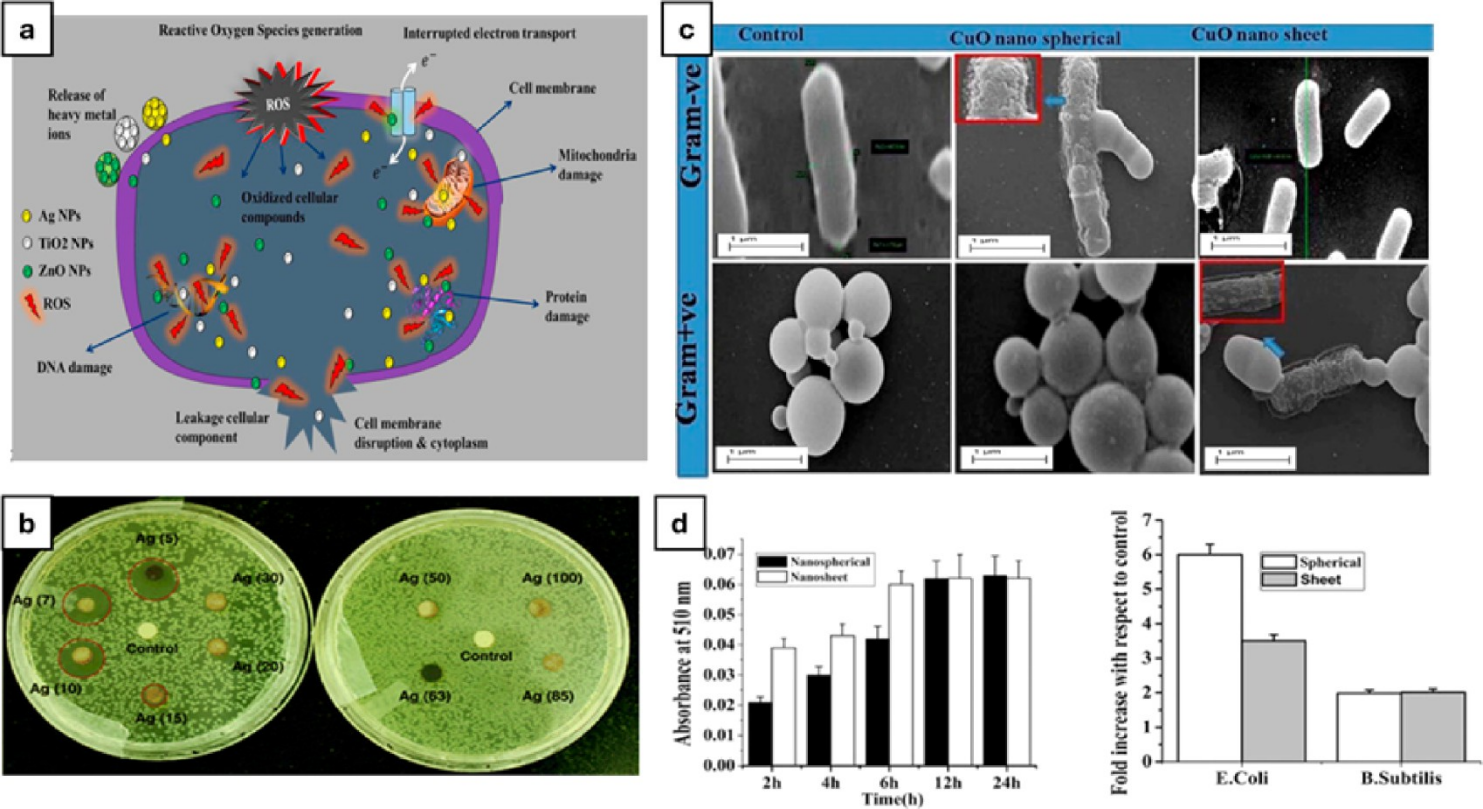
(a) General overview of the antimicrobial mechanisms that nanoparticles can produce. Reprinted with permission from ref 234. Copyright 2018 Elsevier. (b) Antimicrobial effect of differently sized AgNPs against *E. coli*. Adapted with permission under a Creative Commons CC BY 3.0 from ref 32. Copyright 2014 Royal Society of Chemistry. (c) SEM images of *E. coli* and *B. subtilis* bacteria in the presence of CuO NPs with varying shapes (spherical and sheet). (d) CuO release from two different shapes of CuNPs, ROS generation by *E. coli* and *B. subtilis* in the presence of differently shaped CuNPs. Reprinted with permission from ref 235. Copyright 2014 Elsevier.

Nanoparticles can interact with the bacterial cells in two major ways: through (1) external interactions with the cellular membranes, leading to adhesion phenomena, or through (2) internal effects mediated through cell penetration and subsequent interactions with cytosolic components. These mechanisms are dependent on several characteristics of the nanoparticles such as their size, shape, concentration, colloidal state, and oxidation state, which can be modulated according to the intended effect. Below, we summarize the major mechanisms reported specifically for silver and copper nanoparticles.

### 6.1 Physico-Chemical Properties and Their Impact on Antimicrobial Activity

Several papers in the literature report the occurrence of size-dependence in the antimicrobial activity of both silver and copper nanoparticles, which is clearly visible in Figure 6b, in which, differently sized AgNPs yield different inhibition halos for the same *E. coli* strain.^234^ Some papers state a size threshold of 50 nm for the nanoparticle diameter for optimal antibacterial activity. Silver nanoparticles with diameters between 10 and 15 nm have been reported as having increased antibacterial activity when compared to larger nanoparticles.^236^ Similarly, smaller copper nanoparticles, with diameters below 20 nm, have been described as more effective than their larger formulations.^237^ A wide variety of authors corroborate the size-dependent activity of nanoparticles regarding their specific antibacterial activity for both Gram-positive and Gram-negative bacteria. It is believed that a smaller nanoparticle size would allow for easier cellular penetration and an easier later interaction with other cytosolic components.^238–240^

Additionally, the shape of the nanoparticles is also an important physical aspect to determine their antibacterial activity, as seen in Figure 6c. CuO NPs in spherical or rod-like shapes yield different effects on both Gram-positive and Gram-negative strains. The shape of the particles can also influence their bioactive mechanisms, as seen in Figure 6d, as differently spherical and sheet-like particles release Cu ions at different rates toward the medium, resulting in different ROS generation. Numerous studies have pointed out that nanoparticles interact with microorganisms in a shape-dependent manner. A higher surface-to-volume nanoparticle ratio is tendentially associated with a higher antibacterial effect.^241^ Not only these, but also variations in active facets and crystal planes of the nanoparticles will also influence their produced effects.^242–246^

The concentration of nanoparticles to which microorganisms are exposed is yet another crucial factor in their antibacterial activity. Similar to other compounds, nanoparticles act following a specific dose–response relationship. This effect is widespread with regard to the type of nanoparticle, being observed in the vast majority of AgNPs and CuNPs reported in the literature.^247–251^ This dose–response is oftentimes proven through the minimal inhibitory concentrations (MIC), minimum bactericidal concentrations (MBC), or through inhibition zones.

Other factors such as the oxidation state and the colloidal state of the nanoparticles also influence their capacity to produce an inhibitory or bactericidal activity against both Gram-positive and Gram-negative bacteria.^252–254^

### 6.2 Interactions with Cell Membranes (Adhesion Mechanisms)

The first interactions nanoparticles have with bacteria are through their external membranes. These interactions are usually mediated by electrostatic interactions between the negatively charged cell walls and membranes and the positively charged nanoparticles. The presence of teichoic acids in Gram-positive bacteria, and lipopolysaccharides (LPS) and outer membrane proteins (OMP) in Gram-negative bacteria provide them with a slight negative surface charge, facilitating the occurrence of interactions.^255–257^

Several effects have been reported as consequences of these NP-bacterial interactions: 1) morphological alteration of the membrane structure; 2) alteration of membrane permeability; 3) depolarization of membrane zones mediated through electrostatic interactions; 4) leakage of cytosolic components; and 5) decreased transport activity.^231^ TEM images have been collected, showing the morphological effects of AgNPs in bacteria, in which it is possible to verify the leakage of cytosolic components to the outer environment, compromising the normal bacterial functioning.^258^

Although similar in the occurrence of adhesion, AgNPs and CuNPs might have specific intrinsic mechanisms and affinities toward specific strains of bacterium types. For example, copper has a stronger affinity toward proteins than lipids, so it should favor adhesion in Gram-positive bacteria due to their higher protein content, more easily disrupting these bacteria.^259^ Conversely, AgNPs appear to have a stronger activity toward Gram-negative bacteria due to the less complex membrane and the presence of LPS on its surface, mediating electrostatic NP–bacteria interactions.

Adhesion mechanisms of NPs to bacterial cell walls are complex and depend on the type of bacteria evaluated; however, these play a big primary role in the antibacterial activity of both AgNPs and CuNPs. Once again, a single mechanism cannot be pinpointed as the major contributor toward antibacterial activity, with this activity being resultant from a combination of these behaviors.

### 6.3 Cell Penetration and Interactions with Cytoplasmic Elements

The second stage of NP–bacteria interaction occurs upon internalization of the NPs in the cytoplasmic environment. The cytosolic environment is a rich and complex medium in which NPs can interact with a variety of biomolecules. However, before reaching said environment, NPs must traverse the complex membrane systems of Gram-positive and Gram-negative bacteria. For Gram-negative bacteria, porins have been reported as playing a key role in the internalization of both AgNPs and CuNPs.^260–262^ For Gram-positive bacteria, it is thought that AgNPs can enter the cytosolic space through disruption of the peptidoglycan layer,^263^ and CuNPs also enter through peptidoglycan disruption mediated ROS generation, causing oxidative stress in the unsaturated fatty acids of phospholipids contained in the cellular membrane and triggering the peroxidation of the unsaturated fatty acids also present in the membrane, morphologically altering the membrane.^235,264^

After internalization, NPs can interact with multiple biomolecules, which can lead to specific mechanisms that contribute to the inhibition of normal cellular machinery functioning. Through the release of Ag and Cu metal ions and their later accumulation, mismetalation phenomena can occur. For example, Cu^2+^ metal ions can replace the iron in the active center of Fe/S cluster proteins, impairing the function of these cluster proteins, essential for several important cellular mechanisms such as DNA replication, sensing, transcription functions, and more.^265^ Ag^+^ metal ions can also bind to thiol groups in active centers of proteins involved in transmembrane ATP generation and ion transport, deactivating them,^266^ or even with ribosomes, leading to their denaturation.^267^

Both CuNPs and AgNPs can also interact with DNA, causing its degradation and/or denaturation, leading to a loss of genetic cellular material. CuNPs have been reported to be able to degrade double-stranded DNA,^268^ similarly to AgNPs, which have been reported as being able to cause irreversible damage to DNA, through strand breaks, Ag^+^ interactions with nucleic acids, intercalation of AgNPs in the DNA strand blocking the progress of the transcription machinery, and DNA denaturation and morphological alteration to a more condensed form.^269,270^

### 6.4 Reactive Oxygen Species (ROS) Generation

Perhaps the most described mechanism for the antibacterial action of nanoparticles is the generation of ROS and the subsequent induction of oxidative stress. The three-dimensional arrangement of both Cu and Ag nanoparticles allows for the release of their metal ion constituents, usually Cu^2+^ and Ag^+^, into the environment. These can take part in a variety of mechanisms that form ROS, such as, for example, Haber-Weiss or Fenton-like reactions, in which highly reactive hydroxyl radicals can be generated (•OH). Other ROS such as hydrogen peroxide (H_2_O_2_), superoxide anion (O_2_^–^), singlet oxygen, and hypochlorous acid (HOCl) have also been reported in the literature as a direct consequence of the presence of copper and silver nanoparticles^231,271^ Multiple studies detail the bioactive mechanisms of ROS generation, not only from an antimicrobial perspective but also as a significant anticancer agent.^272–274^ As ROS generation can also be modulated through the shape and size of the produced materials, current research in this field is focused on optimization of the morphological properties of nanoparticles in order to enhance the ROS formation mechanisms.

Regarding AgNPs, the mechanism of antimicrobial activity mediated by ROS production is not yet fully understood. However, it is believed that a combination of factors derived from ROS production occurs in a synergistic manner to produce inhibitory or bactericidal effects in bacteria. Some of the reported mechanisms involve the binding of Ag^+^ metal ions to the bacterial membrane, which allows for an influx of oxygen into the cytosolic environment and a subsequent increase in oxidative stress, which directly impairs the normal functioning of the cellular mechanisms.^275–277^

The production of ROS, mediated by AgNPs, can directly affect membrane lipids, proteins, and DNA, causing macromolecular oxidation phenomena and the disruption of normal cell functions. This process is associated with the disruption of the electron transport chain and a significant increase in oxidative stress, evident through variation in the levels of reactive nitrogen intermediates (RNI) and the reduction of glutathione to its oxidized form of glutathione disulfide. The combination of these mechanisms provides a combined approach that integrates both cytotoxic and genotoxic effects, resulting in a combined antibacterial method. These mechanisms are also effective against antibiotic-resistant bacterial strains and aid in hindering the propagation of resistance mechanisms. As ROS have a wide variety of targets, it is harder for bacteria to develop resistance mechanisms that are effective against all these, thus slowing the propagation of novel resistance mechanisms.^278^

Similarly to what is reported in the literature for AgNPs, CuNPs can also produce antibacterial activity through the production of ROS. Once again, these mechanisms are not fully clarified and understood; however, several approaches have been proposed in the literature.^230^ A proposed mechanism, like what was already explained for AgNPs, is through the oscillation between oxidative states, producing O_2_^–^• and OH•, which can lead to lipid peroxidation, DNA, and protein oxidation.

Another reported mechanism for CuNP-mediated ROS generation is through the corrosion of the metallic core of the nanoparticle. This approach is commonly referred to as the “Trojan Horse” approach, in which the nanoparticle is incorporated into the cytosolic environment and only then produces its inhibitory activity. The natural oxidation of copper can also occur, releasing copper ions into the medium and mediating even more of the production of ROS. Additionally, with an increase in the amount of ROS in the cytosolic medium, it will also increase the oxidation of the nanoparticles, leading to an exponential trend.^279^

## 7 Antimicrobial Synergetic Effects of AgCu NPs

Section 6 of this review details specific antimicrobial mechanisms of action mainly derived from Ag- and Cu-based nanomaterials, as these have been extensively reported in the literature. Although not as extensively studied as their monometallic counterparts, AgCu-based nanomaterials tend to present an improved overall antimicrobial activity toward Gram-positive and Gram-negative bacteria.^280^ Additional variables can be taken into account when considering the antimicrobial effects of the bimetallic materials, such as the metal ratios present in them and their specific three-dimensional arrangements.

A recurrent trend regarding Ag:Cu ratios present in the nanomaterials is that a higher Ag percentage tends to perform better than those containing higher Cu percentages, possibly due to the increased release of Ag ions into the medium in nanoalloy materials, with Ag atoms being more easily oxidized in the presence of Cu atoms, subsequently increasing their release into the medium and resulting in a higher antimicrobial profile.^158^

Another possible mechanism that explains the improved antimicrobial profile of bimetallic nanomaterials is related to a multitarget approach. Biological components, such as enzymes or proteins, that are more susceptible to a single metal will also be affected by the bimetallic material, thereby increasing their potential biological targets.^160^

Although the release rate of Ag and Cu ions into the medium appears to be one of the major mechanisms of action studied for this type of bimetallic nanomaterial, a combination of all of the previously mentioned mechanisms is still in place upon the release of the ions into the medium. Once again, the antimicrobial profile of the material does not correlate with a single mechanism of action but through a combination of several mechanisms, which can be more or less prevalent, dependent on the biological context.^281–283^

Further studies must be performed not only in understanding the proper mechanisms for the release of the metal ions into the medium, which appear to be dependent on the morphological characteristics and tridimensional arrangement of the nanomaterial, but also in understanding the direct biological implications that this release will have on a cellular environment, whether in a eukaryotic or prokaryotic cell. Through these studies, further optimization of the synthetic protocols can be performed to increase the antimicrobial profile of these materials. Regardless, a clear trend is demonstrated in the literature, determining that the bimetallic nanoparticles present an increased antimicrobial profile compared to their respective monometallic counterparts.

## 8 Applications of Nanoparticle Doped-Polymers

The previous points have detailed synthetic strategies for both nanomaterials and polymeric matrices, as well as the specific antimicrobial mechanisms of action that these materials can present. All of those are of utmost importance to fully understand the potential applications of such materials. Several other reports have already detailed the specific applications of metallic nanoparticles and their uses in areas as diverse as biosafety, healthcare, or dentistry for both silver and copper nanoparticles.^8,34,284–286^ As such, this review will focus on the specific applications of the silver and/or copper nanoparticle-doped polymeric materials.

Through the incorporation of the bioactive nanoparticles onto a solid-supported matrix, the applicability of the nanoparticles increases drastically. It allows for the preparation of antimicrobial surfaces or coatings for high-risk areas, food packaging, and bioactive clinical materials, among others. Below, some of these specific applications are detailed, separated by their specific type of application, and focusing on the specific bioactive synergistic effect derived from the incorporation of nanoparticles onto these polymeric matrixes.

### 8.1 Healthcare Applications

Healthcare applications can be widely vast, ranging from direct therapeutic approaches to bioactive surface materials or even tissue engineering, with effects that might be related to morphological cellular alterations, as can be seen in Figure 7a. Through confocal microscopy techniques, it is clear to see a significant morphological alteration, in the presence of the nanocomposite material, leading to cell death.^287^ One of the most widely reported uses for antimicrobial polymers such as those described in this review is the production of antimicrobial coatings.^288–290^

**Figure 7:**
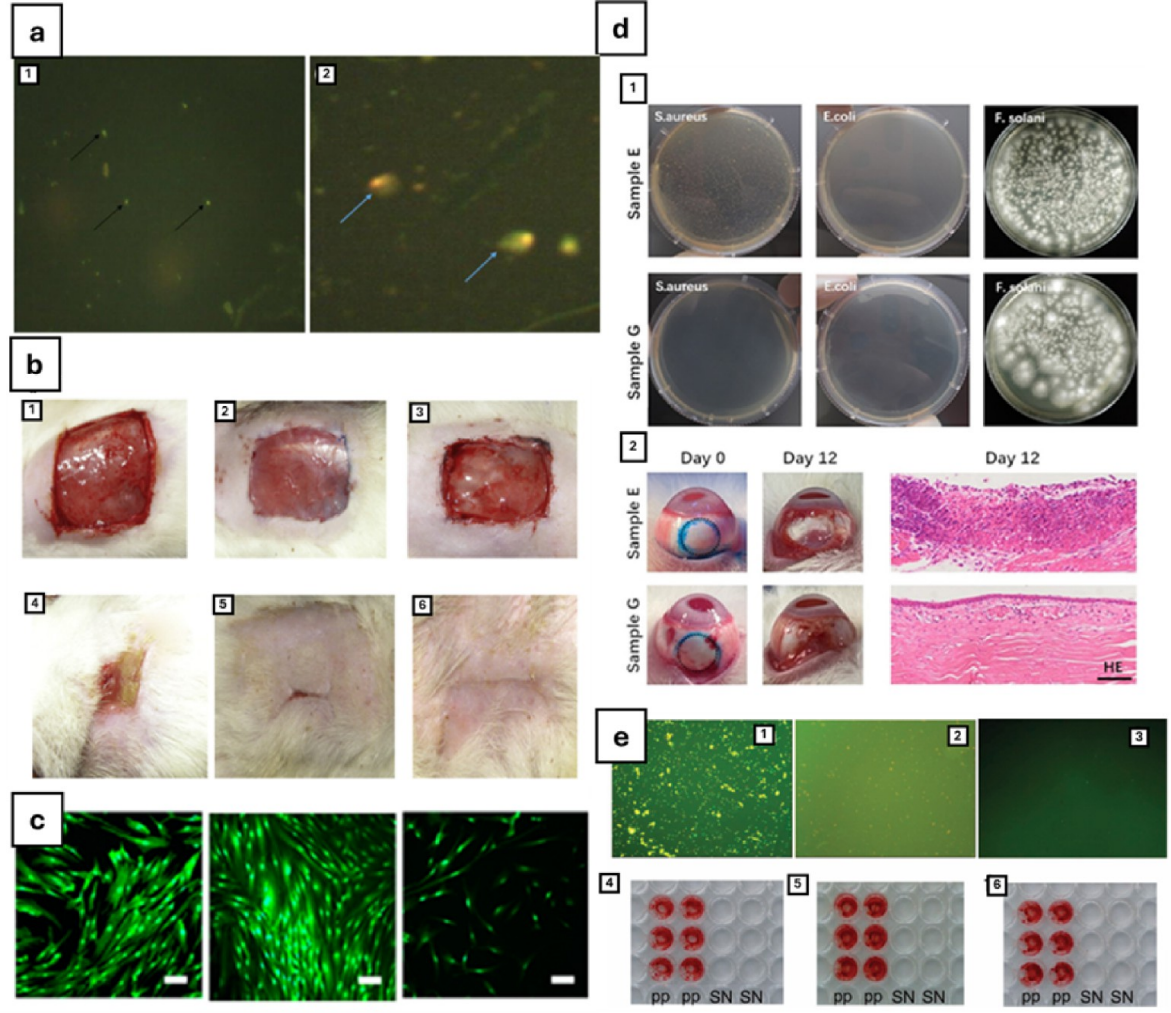
(a) Morphological alterations in the nucleus of Caco-2 cells in the presence of the nanocomposite. Reprinted with permission from ref 287. Copyright 2022 Elsevier. (b) Wound healing progression at several days of healing in the presence of the silver nanocomposite material. (Day 0) 1, 2, 3 – wound controls; (Day 16) 4 – control; 5, 6 – wound healed in the presence of the nanocomposite materials. Reprinted with permission from ref 291. Copyright 2012 Elsevier. (c) Confocal microscopy images of HDF cells treated with AgNPs (right). Reprinted with permission from ref 292. Copyright 2015 American Chemical Society. (d) 1 – Antibacterial activity of the nanocomposites (containing AgNPs) in response to various bacterial strains. 2 – Ocular transplant of the nanocomposites onto the conjunctival defective tissue. Reprinted with permission from ref 293. Copyright 2023 Elsevier. (e) Antimicrobial activity of the nanocomposite material (containing AgNPs) against *S. epidermis*. Biofilm formation assays. Reprinted with permission from ref 294. Copyright 2010 American Chemical Society.

One such example is reported by Patil et al.,^295^ in which an AgNP-doped polystyrene with remarkable antimicrobial activity against *Escherichia coli* and *Staphylococcus aureus* was synthesized. It is proposed that this antimicrobial activity might be formed through the generation of free radicals, and interestingly, it is also modulated by the film thickness. A thickness of 10–15 nm presented a higher antimicrobial activity than the tested counterpart, only 5 nm. These differences in results might be related to the amount of silver released to the medium, which was determined to be a continuous silver release through at least 3 days of contact. This assay demonstrates the effect that the physical characteristics of the produced materials can have on the final application of the material and furthermore reinforces the need for attention to detail necessary for the synthesis of the materials.

Fuchs et al.^296^ present yet another example of the synthesis of a long-term silver nanoparticle-doped antimicrobial material, usable to produce materials and surfaces in a clinical environment. Once again, the controlled and time-dependent release of silver ions is critical for the antimicrobial activity of the material. This study is also interesting as it proposes another mechanism of action for silver release, not directly from the polymer to the medium in a more abrupt way, decreasing the overall total amount of silver in the matrix, but through a more gradual dissolution and presence of silver ions in the hydration interface of the polymer with the medium. Through a somewhat conserved presence of silver ions in this interface, a long-term antimicrobial effect can be produced in a synergistic approach between the nanoparticles and their polymeric counterparts. Additionally, the entrapment of nanoparticles onto a solid-supported matrix will also decrease their overall toxicity to humans,^297^ producing a more directed approach to antimicrobial effects.

Through the production of antimicrobial polymeric surfaces, a more direct therapeutic approach can also be taken. Taheri et al.^292^ (Figure 7c) have reported the production of thin plasma polymer films containing silver nanoparticles that present significant antibacterial activity. However, these films were also tested with regard to their possible generation of inflammatory responses, demonstrating a reduced response. This additional aspect allows for the development of another approach, which is the incorporation of these materials into the production or coating of biomedical devices such as catheters or wound dressings.

This wound dressing approach is yet another popular healthcare application reported by various authors.^298–302^ To this end, it is very important to evaluate not only the antimicrobial effects that the polymers produce, but it is equally important to assess the nontoxic nature of the material toward mammalian cells in order to prevent toxic effects toward humans.^294^ As an example, Arockianathan et al.^291^ have successfully demonstrated that the presence of nanoparticle-doped films has improved healing time patterns. These films were based on an alginate and starch monomeric basis and were doped with silver nanoparticles, ensuring not only a biocompatible but also a sustainable profile of the polymer. To perform a well-documented evaluation of the wound healing properties of the materials, antibacterial studies were complemented by histological and planimetric evaluations. A complete healing of the wounds was observed in only 16 days in the presence of the polymeric material, compared to the 18 days it took for the wound to fully heal in the control groups, as seen in Figure 7b. As such, these results are indicative of the potential of nanoparticle-doped polymeric materials to be used in therapeutic approaches.

These materials also show promise from a tissue engineering viewpoint. One example is the use of AgNPs/Silk peptide (SP)-doped PLA films, as reported by Zhang et al.,^293^ as they can be modulated to act as conjunctival replacement tissue, with results pointing toward a good transplantation material, with improved antimicrobial activity against *E. coli*, *S. aureus*, and *F. solani*, as shown in Figure 7d. The presence of silk peptide promotes the overall healing process with the addition of AgNPs acting as an additional antibacterial vector within the matrix. In the tissues treated with the AgNPs-doped material, no infectious bacterial response was verified, thus, corroborating the use of these materials as promising tissue engineering vectors. Additionally, these materials also present promising results in hindering the formation of biofilm structures against strains such as *S. epidermis*, as shown in Figure 7e.^294^

A drug-delivery approach has also been tested in such polymers, for example, through the synthesis of a collagen/chitosan blended polymer doped with not only AgNPs but also ibuprofen for topical application in wounds, both infected and noninfected,^303^ and also the delivery of curcumin to cancer cells, through a curcumin/AgNP-doped modified chitosan polymer.^287^ Sensing probes have also been developed, dependent on AgNP-doped polymers specifically for skin cortisol,^304^ as well as UV–vis light barriers to act as protective membranes to prevent against sun damage.^305,306^ Regardless of these applications, the basal antimicrobial activity of the polymers is present as a constant throughout the materials described. These studies indicate the versatility that nanoparticle-doped polymers can have in a clinical setting, easily modulated by the incorporation of additives, whether compounds or nanoparticles, in a polymeric matrix.

### 8.2 Food-Related Applications

The antimicrobial activity of polymers mediated through the incorporation of nanoparticles onto their composition has an interesting application in the development of food packaging materials, as reported through various studies.^307^ The inherent antimicrobial properties of these matrices help in increasing the shelf life of food items as bacterial proliferation is one of the major causes of food spoilage (Figure 8). Through the control of microorganism proliferation rate, food items will spoil slower, reducing food waste and decreasing the probability of consumption of contaminated items. An example might be seen in Figure 8a,b, where PLA and blended PLA/MPE films doped with AgNPs were evaluated for their antimicrobial activity, demonstrating a superior bioactive profile than the nondoped polymers tested. Additionally, these films were also employed as food packaging for strawberries, with results pointing to a better conservation than commercial films and nondoped polymers. Strawberries maintained their properties even after 7 days in a better conservation state than the other tested films. These results are derived from the antimicrobial activity of the AgNPs and the synergistic effects derived from their incorporation onto the polymeric matrix, allowing for a directed application.^308^

**Figure 8:**
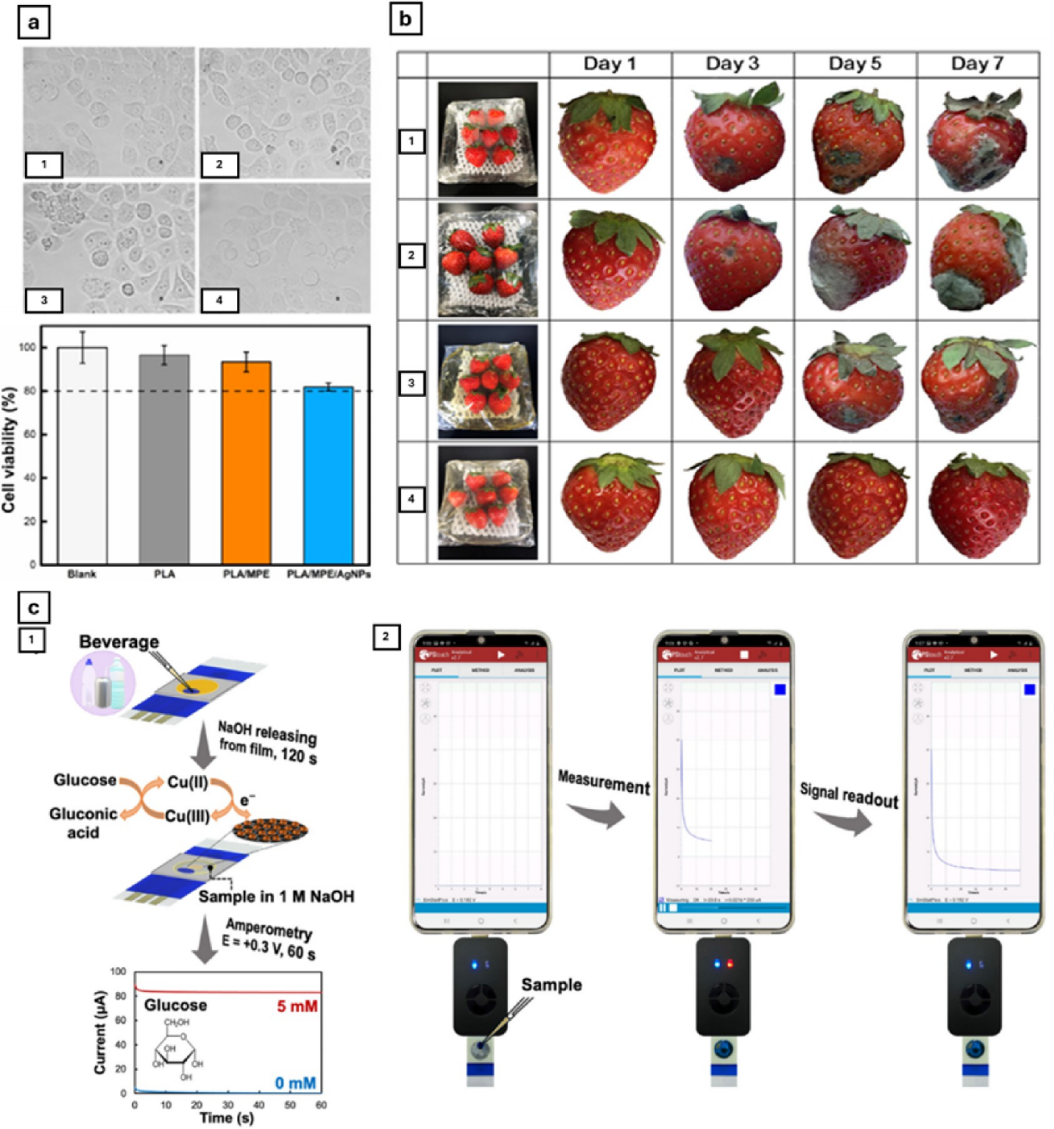
(a) Cell toxicity of the thin films: a – blank; b – PLA film; c – PLA/MPE film; d – PLA/MPE/AgNP film. (b) Photographs showing the effect of the films on strawberry preservation: (1) PE cling film, (2) PLA film, (3) PLA/MPE film, and (4) PLA/MPE/AgNP film. Reprinted with permission from ref 308. Copyright 2021 Elsevier. (c) Schematic diagram of the glucose detection system (1) and photograph of the measurement procedure based on the one-step glucose sensor (2). Adapted with permission under a Creative Commons CC BY 4.0 from ref 309. Copyright 2023 Elsevier.

Regardless of the promise that these materials can demonstrate for this specific application type, the migration of the additives, and most importantly nanoparticles in the matrix toward the food items is an important aspect to consider for these matrixes.^310^ These must still allow for the occurrence of antimicrobial activity; however, the release rates must be controlled and should be optimized to be the minimum concentration that can produce a sufficient antimicrobial effect over a long time period.^311^ Food safety standards are strict with the amounts of materials that can be released into food items in packaging materials, and as such, the produced materials must comply with these regulations. Regardless, through control of these aspects, several advantages can be derived from the use of nanoparticle-doped polymers as bioactive food packaging materials.

Interestingly, most of the reported polymers to address this issue are based on biocompatible monomeric units such as polylactic acid,^308^ chitosan,^312,313^ gelatin,^314,315^ agar,^184^ PVA,^316,317^ or even cellulose.^318^ Overall, all these matrixes present a significant inherent antimicrobial activity that can be extrapolated toward a biofunctional food packaging material. Additionally, blended materials have also been reported through a combination of various polymeric materials, enhancing their mechanical properties such as water permeability or tensile strength. Examples are blends such as PVA/starch/glycerol,^319^ gelatin/cellulose,^320^ chitosan/pectin,^321^ or even PVA/montmorillonite/starch.^322^

In another approach, the development of food-related sensors has also been reported using polymeric materials doped with nanoparticles. For example, Yarynka et al.^323^ have developed a sensor to determine the presence of zearalenone, a fungus-produced mycotoxin that can threaten human health, particularly in young children. For determining the presence of this toxin in flour samples, a nanoparticle-doped molecular imprinted polymer (MIP) was developed through a radical polymerization technique. This material was designed to specifically determine the presence of the toxin selectively with a limit of detection (LOD) of 5 ng/mL and a linear range of quantification from 5 to 25 μg/mL, allowing for an accurate toxin quantification. Preechakasedkit et al.^309^ have also developed a nanoparticle-doped polymeric material containing copper nanoparticles to allow for the determination of glucose levels in food items. This technique is meant to act as a confirmation tool to quickly and easily corroborate the glucose levels described on the food labels of beverages, and a detailed overview of the process is detailed in Figure 8c. This matrix was composed of a NaOH/PVA/PEO blended matrix doped with CuNPs and allows for the glucose detection based on a smartphone-based potentiostatic measurement, with a LOD of 0.06 μM and a linear range of detection of 0.01 to 7 mM.

### 8.3 Others

Other approaches have also been reported for nanoparticle-doped polymers, such as those reported in this review. A brief listing of those applications is reported here to further demonstrate the potential of these materials to a variety of applications, not only related to a biological viewpoint but also in more industrial applications.

These can be as varied as the use of multilayered polymeric material doped with AgNPs to be used as an electronic tongue that allows for the differentiation between the basic flavors through impedance measurements.^324^ Another interesting strategy includes the use of nanoparticle-doped polymers as flexible electronic devices,^325–327^ for thermal sensitive applications,^328^ as enhancers of photostability of electronic devices^329^ and of electric conductivity,^330^ as heat insulator materials,^331^ or even as shields against electromagnetic interference.^332^

Environmental applications have also been developed and are well-reported in the literature, ranging from hydrogen sulfide gas^333^ or universal Hg^334^ sensors to photocatalytic materials with significant antimicrobial activity^335^ or even as materials for the removal of organic dyes^336^ and drugs^337^ using photo-oxidation mediated by AgNP-doped polymers or even desalinization materials.^338^

## 9 Future Perspectives

Metallic nanoparticles, particularly silver, copper, and their bimetallic hybrids, have emerged as powerful antimicrobial agents with broad applications to address the rise of antimicrobial resistance. Their ability to disrupt microbial membranes, generate reactive oxygen species, and interfere with cellular machinery positions them as promising tools in both therapeutic and preventive domains. When embedded within polymeric matrices, these nanoparticles gain enhanced mechanical stability, sustained release profiles, and improved usability across diverse environments, ranging from clinical settings to food safety applications.

This review summarizes the major synthetic routes for silver (AgNPs), copper (CuNPs), and silver–copper (AgCuNPs) nanoparticles, highlighting the methods used to incorporate these nanomaterials into polymeric matrices. These approaches enable the production of homogeneous, well-dispersed materials with a wide range of applications, from healthcare to industrial uses. A key advantage of such materials is the ease with which their properties can be modulated to suit specific applications. By ensuring compatibility between nanoparticles and the monomeric components of polymeric matrices, multiple combinations can be optimized for targeted outcomes. Among the various applications, the antimicrobial effects of these materials are of particular interest. These nanoparticles demonstrate significant activity against a broad spectrum of microorganisms, especially pathogenic bacteria, which remains a focal point in ongoing research. However, while the antimicrobial properties of AgNPs, CuNPs, and AgCuNPs are well-documented, further studies are essential to fully understand their mechanisms of action and interactions with polymeric matrices.

Despite the promising antimicrobial activity, key challenges, such as scaling up production, ensuring biocompatibility, long-term stability, and cytotoxicity, must be addressed to fully realize their potential and safely integrate them into real-world applications. The absence of unified regulatory guidelines for nanoparticle-doped polymers complicates their placement in healthcare and consumer products.

Further studies are also required to assess the impact of these materials on mammalian cells to ensure their safety for use in specific applications and their potential human impact. Moreover, while various synthesis routes, such as chemical, physical, and biological methods, allow for the flexible fabrication of nanoparticles, the choice of method significantly influences their size, morphology, and functional performance.

The next generation of antimicrobial nanocomposites will be defined not only by efficacy but also by intelligence, sustainability, and translational readiness. Among the most promising directions is the rational design of bimetallic nanoparticles, particularly AgCu systems, with controlled architectures (e.g., core–shell, alloy, Janus) that control synergistic effects and enable tunable ion release. Fine control over particle size, shape, crystallinity, and metal ratios will allow researchers to optimize the antimicrobial potency while minimizing off-target toxicity. To this end, the implementation of advanced synthetic approaches such as flow chemistry, microwave-assisted methods, and bioinspired green synthesis will be pivotal in achieving reproducibility, scalability, and environmental viability.

Future research should prioritize the integration of these nanoparticles into biodegradable and biocompatible polymer matrices, creating functional materials capable of degrading safely after use. Additionally, rigorous *in vivo* and long-term studies are needed to evaluate biodistribution, biodegradation, and potential immunogenicity, particularly for medical and food-contact applications. Such efforts are vital to unlocking the translational potential of these nanocomposites as next-generation antimicrobial materials for a wide array of real-world applications in healthcare, industry, and beyond. With continued optimization and mechanistic insight, these systems may offer safer and more effective tools to combat microbial threats.

Another major frontier lies in the development of multifunctional and stimuli-responsive nanocomposites. By incorporating elements such as pathogen-responsive triggers, pH- or temperature-sensitive release mechanisms, or colorimetric indicators, these materials can evolve from passive barriers to active intelligent systems. Their application in chronic wound care, implantable medical devices, and smart packaging could revolutionize how antimicrobial protection is delivered and monitored. Furthermore, expanding their functional versatility to include antiviral, antifungal, or even antiinflammatory properties could enhance their utility in addressing broader public health challenges.

Equally critical is the establishment of robust regulatory and safety frameworks. Despite encouraging *in vitro* results, the absence of standardized toxicological and environmental evaluations limits the path to commercialization. Developing harmonized international protocols for assessing nanoparticle–polymer systems, covering ecotoxicity, occupational exposure, and long-term human health effects, will be essential to ensure both efficacy and public safety.

Finally, the integration of artificial intelligence (AI) and machine learning (ML) into nanomaterial discovery holds a transformative potential. Predictive models informed by experimental and computational datasets can enhance the design of materials with optimized performance and safety profiles.

## Abbreviations

AgCu NPs: silver copper nanoparticles
AgNPs: silver nanoparticles
AMR: antimicrobial resistance
CTAB: cetyltrimethylammonium bromide
CuNPs: copper nanoparticles
GMP: Good Manufacturing Practices
LED: light-emitting diode
LOD: limit of detection
LPS: lipopoylssacharides
LSPR: localized surface plasmon resonance
MIP: molecular imprinted polymer
OMP: outer membrane proteins
PEG: polyethylene glycol
PLA: pulsed laser ablation
PLD: pulsed laser deposition
PMMA,: polymethyl methacrylate
PPL: pulsed plasma in liquid
PVA: poly­(vinyl alcohol)
PVD: physical vapor deposition
PVDF: polyvinylidene fluoride
PVP: polyvinylpyrrolidone
RNI: reactive nitrogen species
ROS: reactive oxygen species
TEM: transmission electron microscopy
USP: ultrasonic spray pyrolysis
UV: ultraviolet
**Vocabulary**
Antimicrobial Resistance (AMR): is a natural selection process in which microorganisms evolve to evade or withstand the bioactive effects of antimicrobial agents as antibiotics, antifungals, etc. This process has become a public health issue due to the exponential growth of resistance mechanisms exacerbated by excessive exposure to these antimicrobial agents.
Nanoparticles (NPs): are materials with dimensions between 1 and 100 nm, which exhibit specific physical, chemical, and bioactive properties, different from those presented by the bulk materials.
Silver Nanoparticles (AgNPs): are metallic nanoparticles composed of silver that exhibit significant broad-spectrum antimicrobial properties. These particles can act through various mechanisms such as by formation of reactive oxygen species and inhibition of DNA replication, among others.
Copper Nanoparticles (CuNPs): are metallic nanoparticles composed of copper, which present significant bioactive and catalytic properties. CuNPs can have a significant broad-spectrum antimicrobial activity.
Silver–Copper Bimetallic Nanoparticles (AgCu NPs): are nanoparticles composed of both silver and copper with enhanced stability and bioactivity from their single-metal counterparts. These particles yield synergistic effects from the combination of both metals, improving the overall efficacy of the nanoparticles.
Polymeric matrix: a structural three-dimensional mesh composed of natural or synthetic polymers in which compounds or nanoparticles can be doped. This integration enables the production of smart, bioactive materials, which enable their application in a variety of areas.
